# The use of environmental data in descriptive and predictive models of vector-borne disease in North America

**DOI:** 10.1093/jme/tjae031

**Published:** 2024-03-03

**Authors:** Hanna D Kiryluk, Charles B Beard, Karen M Holcomb

**Affiliations:** Division of Vector-Borne Diseases, Centers for Disease Control and Prevention, 3156 Rampart Road, Fort Collins, CO 80521, USA; Colorado School of Public Health, Colorado State University, Sage Hall, Campus Delivery 1612, Fort Collins, CO 80523, USA; College of Veterinary Medicine and Biomedical Sciences, Colorado State University, 1601 Campus Delivery, Fort Collins, CO 80523, USA; Division of Vector-Borne Diseases, Centers for Disease Control and Prevention, 3156 Rampart Road, Fort Collins, CO 80521, USA; Division of Vector-Borne Diseases, Centers for Disease Control and Prevention, 3156 Rampart Road, Fort Collins, CO 80521, USA

**Keywords:** vector-borne disease, North America, modeling, forecasting, climate

## Abstract

Vector-borne disease incidence and burden are on the rise. Weather events and climate patterns are known to influence vector populations and disease distribution and incidence. Changes in weather trends and climatic factors can shift seasonal vector activity and host behavior, thus altering pathogen distribution and introducing diseases to new geographic regions. With the upward trend in global temperature, changes in the incidence and distribution of disease vectors possibly linked to climate change have been documented. Forecasting and modeling efforts are valuable for incorporating climate into predicting changes in vector and vector-borne disease distribution. These predictions serve to optimize disease outbreak preparedness and response. The purpose of this scoping review was to describe the use of climate data in vector-borne disease prediction in North America between 2000 and 2022. The most investigated diseases were West Nile virus infection, Lyme disease, and dengue. The uneven geographical distribution of publications could suggest regional differences in the availability of surveillance data required for vector-borne disease predictions and forecasts across the United States, Canada, and Mexico. Studies incorporated environmental data from ground-based sources, satellite data, previously existing data, and field-collected data. While environmental data such as meteorological and topographic factors were well-represented, further research is warranted to ascertain if relationships with less common variables, such as oceanographic characteristics and drought, hold among various vector populations and throughout wider geographical areas. This review provides a catalogue of recently used climatic data that can inform future assessments of the value of such data in vector-borne disease models.

## Introduction

In recent years, the incidence and burden of vector-borne diseases have increased dramatically. The number of reported cases has more than tripled in the United States, from 27,388 cases in 2004 to 96,075 cases in 2016 (Rosenberg et al. 2018). However, reported cases underestimate the true burden of vector-borne diseases (Beard et al. 2019). For example, approximately 30,000 cases of Lyme disease are reported annually in the United States (Centers for Disease Control and Prevention, 2022). However, using commercial insurance claims data, the Centers for Disease Control and Prevention (CDC) estimated 476,000 patient diagnoses and treatments of Lyme disease in the United States occurred annually from 2010 to 2018 compared to a similarly conducted estimate of 329,000 annual diagnosed cases from 2005 to 2010 (Kugeler et al. 2021).

Weather events and climate patterns are noteworthy factors that influence vector populations and disease distribution and incidence. Temperature influences tick questing behavior, thus impacting the likelihood of host acquisition (Ogden et al. 2021). Temperature also affects tick developmental rates from one life stage to the next, with faster developmental rates occurring at warmer temperatures (Ogden et al. 2004, Ogden and Lindsey 2016). Mosquitoes are also sensitive to weather events and climate patterns. Precipitation patterns and the resultant pooling of water contribute to the larval development of mosquitoes. Temperature influences developmental time and overwinter survival rates (Alto and Juliano 2001, Yang et al. 2008, Rochlin et al. 2013). Changes in weather trends and climatic factors can thus alter seasonal vector activity, pathogen development, and host demographic processes, consequently reshaping pathogen distribution and introducing diseases to newly susceptible areas. These shifts have been documented in recent years (Fouque and Reeder 2019).

The global temperature is on the rise, with an approximate 2°F increase in global average surface temperature since the preindustrial period (1880–1900) (Lindsey and Dahlman 2023), and with 2016 and 2020 as the 2 warmest years on record (Greene and Jacobs 2021, NOAA 2021). Most vectors are ectotherms, so it is anticipated that vector abundance, survival, and feeding may increase in many regions in response to rising temperature, along will pathogen development within the vector (Rocklöv and Dubrow 2020). However, these life history traits exhibit unimodal relationships with increasing temperature such that above a thermal optimum, rates decrease (Shocket et al. 2020). Therefore, the change in pathogen transmission rates with increased temperatures are not linear and may be hard to fully predict. Extreme weather events are also escalating, with high precipitation events increasing in intensity and frequency across the United States since 1901 (U.S. Global Change Research Program 2017). Changes in incidence and distribution of disease vectors possibly linked to climate change have already been documented. In Canada, the incidence of endemic mosquito-borne diseases has increased by approximately 10% throughout the last 20 years. Invasive *Aedes* species mosquitoes were first identified in southern Ontario in 2016 as the species moved further north and have been repeatedly documented in the years since (Ludwig et al. 2019). In 2021, Arizona experienced the largest local outbreak of West Nile Virus in US history, potentially associated with a wetter-than-average Southwest monsoon season (Holcomb 2022). Tools to prepare for and combat these changes are of utmost priority.

Forecasting and modeling efforts contribute to the development of such predictive tools. Modeling can supplement traditional surveillance methods both for predicting changes in vector and vector-borne disease distribution related to climate change and for optimizing disease outbreak preparedness and response. These modeled relationships can lead to a better understanding of the relationship between vector-borne disease occurrence and climate change, as estimating the potential geographic range of a species involves characterizing its optimal environmental conditions and then identifying where the optimal conditions exist or are projected to exist (Fischer et al. 2011, Caminade et al. 2012, Estrada-Peña et al. 2012). The purpose of this scoping review was to catalog the use of environmental data in descriptive and predictive models of vector-borne disease prediction in North America from 2000 to 2022.

## Materials and Methods

This review is a comprehensive, but not exhaustive, compilation of published literature that used environmental data to describe or forecast vector-borne diseases or risks in North America.

### Inclusion Criteria

English language journal articles pertaining to North America and published from 1 January 2000 to 2 June 2022 were eligible for inclusion. For the purpose of this review, North America included the United States, Mexico, Canada, and the associated US territories. In addition to Puerto Rico and the U.S. Virgin Islands, we included American Samoa, Guam, and the Northern Mariana Islands, even though not geographically part of North America. Only journal articles written in English were eligible for inclusion due to the native language of the authors and the widespread prevalence of the language throughout the study region. We chose the year 2000 as the starting point because it marked the turn of a new century, and we assumed that any important information published before this date would continue to be present in more current publications. Reviews, books, and conference papers that focused on primary research published in peer-reviewed journals were excluded from the analysis.

### Sources of Information

We searched MEDLINE through PubMed and Web of Science Core Collection databases. We selected these databases due to their focus on physical and life sciences and public health. We searched MEDLINE through PubMed on 1 June 2022 and Web of Science Core Collection on 2 June 2022.

### Search Terms

We used the following character string to conduct database searches: [(disease*) AND (human*) AND (model* OR forecast* OR predict*) AND (vector* OR mosquito* OR flea* OR tick*) AND (weather OR climate) AND (“North America” OR “United States” OR Canada OR Mexico) NOT (Europe OR Asia OR Africa OR “South America” OR Australia)].

We used the same character string for each database, and searches were conducted under “All Fields.” Restrictions on all searches included the date of publication (1 January 2000 to present) and the language (English). We removed duplicate results and all reviews, books, and conference papers using Zotero version 6.0.9 (Corporation for Digital Scholarship, Vienna, Virginia, United States).

### Selection of Journal Articles

After removing duplicate results, we screened the remaining citations to identify the relevant journal articles for inclusion in the review. We completed this in 2 phases. During the first phase, we evaluated titles and abstracts in Zotero and excluded journal articles that were evidently outside the scope of the review. This included articles pertaining to geographic regions outside North America, animal and plant diseases that lack human health implications, and aspects of vector-borne disease unrelated to modeling and climate. During the second phase, we obtained full versions of the remaining articles and located the relevant information required for inclusion in the articles. Relevant articles were those that fit the geographic criteria, focused on a zoonotic vector-borne disease with human health implications, included climate/environmental data in the model, and contained a component of disease prediction (e.g., anticipated geographic expansion of a vector population, determination of emerging areas of disease risk, and best predictive factors of disease risk or vector abundance).

### Data Logging

We used a Google Sheets (Google, Mountain View, California, United States) spreadsheet to log relevant data from each article, allowing authors to collaborate simultaneously on the same document. We included the following information in the spreadsheet (Supplementary Table S1): article identifiers (i.e., title, journal, author, and year of publication); study descriptors (i.e., purpose/goal, disease/pathogen of interest, vector of interest, geographic location, and main findings); and model characteristics (i.e., population model, model category, and environmental data source(s)).

Modeled populations included vectors, humans, and reservoir hosts or sentinels. Examples of sentinel species include chickens (West Nile virus disease; Langevin et al. 2001) and domestic dogs (Lyme disease; Lindenmayer et al. 1991). Wild rodents are an example of reservoir hosts for plague (Gage and Kosoy 2005).

Model categories were assigned based on the primary model that was used to predict vector-borne disease distribution (Table 1). Predictions that incorporated equal use of more than one type of model were assigned to the “Multiple Models” category.

**Table 1.** Definition of categories used to describe model types of included literature

Model category	Definition
Compartmental/deterministic/differential equations	Models tracking the movement of the study population through compartments, like disease states, using differential equations.
Ecological niche modeling/habitat suitability	Models that use associations of environmental conditions with vector presence or absence to predict the suitability of the habitat in other locations. Examples of algorithms used include Maximum Entropy (MaxEnt), Genetic Algorithm for Rule-Set Prediction (GARP), and Raster Spatial Model.
Individual/agent-based	Models that simulate the interactions of individual agents.
Machine learning	Models that use algorithms to determine patterns and/or make predictions include random forests, neural networks, classification trees, and boosted classification trees.
Qualitative	Models that utilized correlations between climate and disease variables and did not perform any statistical testing.
Regression	Models that describe the relationship between one or more independent variables and a response include linear, logistic, Poisson, generalized linear, generalized additive models, and one-way ANOVA.
Risk assessment metrics/correlations/empirical models	Models based on correlations of predictors (e.g., climatic factors, vector indices) with vector-borne disease transmission to predict the risk of disease.
Ross-McDonald/R_0_/vectorial capacity	Mathematical equations based on the one proposed by McDonald relate a series of biological features of vectors to approximate the magnitude of vector-borne disease transmission risk (Macdonald 1956).
Spatial analyses	Models that use spatial data and associated statistics to identify relationships.
Spatio-temporal analyses	Models that accounted for both the marginal and joint spatial and temporal impacts of covariates on the prediction, often through a hierarchical Bayesian approach.
Survival analysis	Models that analyze the expected duration of time until an event occurs.
Time series analysis	Models that use data arranged with respect to time to identify trends and residuals used to forecast future events.

Environmental data sources were compiled from all 205 relevant journal articles (Supplementary Text S2). If the data did not come from a database, it was noted if it came from experimental conditions, fieldwork, or other literature sources. Field work related to the collection of data (e.g., infection prevalence, host or vector abundance, weather observation) from locations in the modeled study area. The data were sorted into 5 main categories: meteorologic, elevation, future climate scenarios/simulations, satellite, and topography. Land use/land cover data were included in the satellite or topography categories based on the indicated data source (e.g., wetland inventories were labeled as “topographic,” and datasets from the Moderate Resolution Imaging Spectroradiometer (MODIS) sensor onboard orbiting satellites were labeled as “satellite”). Additional types of environmental data documented throughout the review were daylight/day length, drought, hurricane, oceanographic, and El Niño–Southern Oscillation (ENSO). Here, we used the term “ENSO variables” to refer to the Southern Oscillation Index that is used to describe El Niño and La Niña years.

### Synthesis of Results

Data cleaning, descriptive statistics, and visualizations were conducted using R statistical programming version 4.1.1 in RStudio version 1.4.1717 (R Core Team, Vienna, Austria).

## Results

**Fig. 1.**
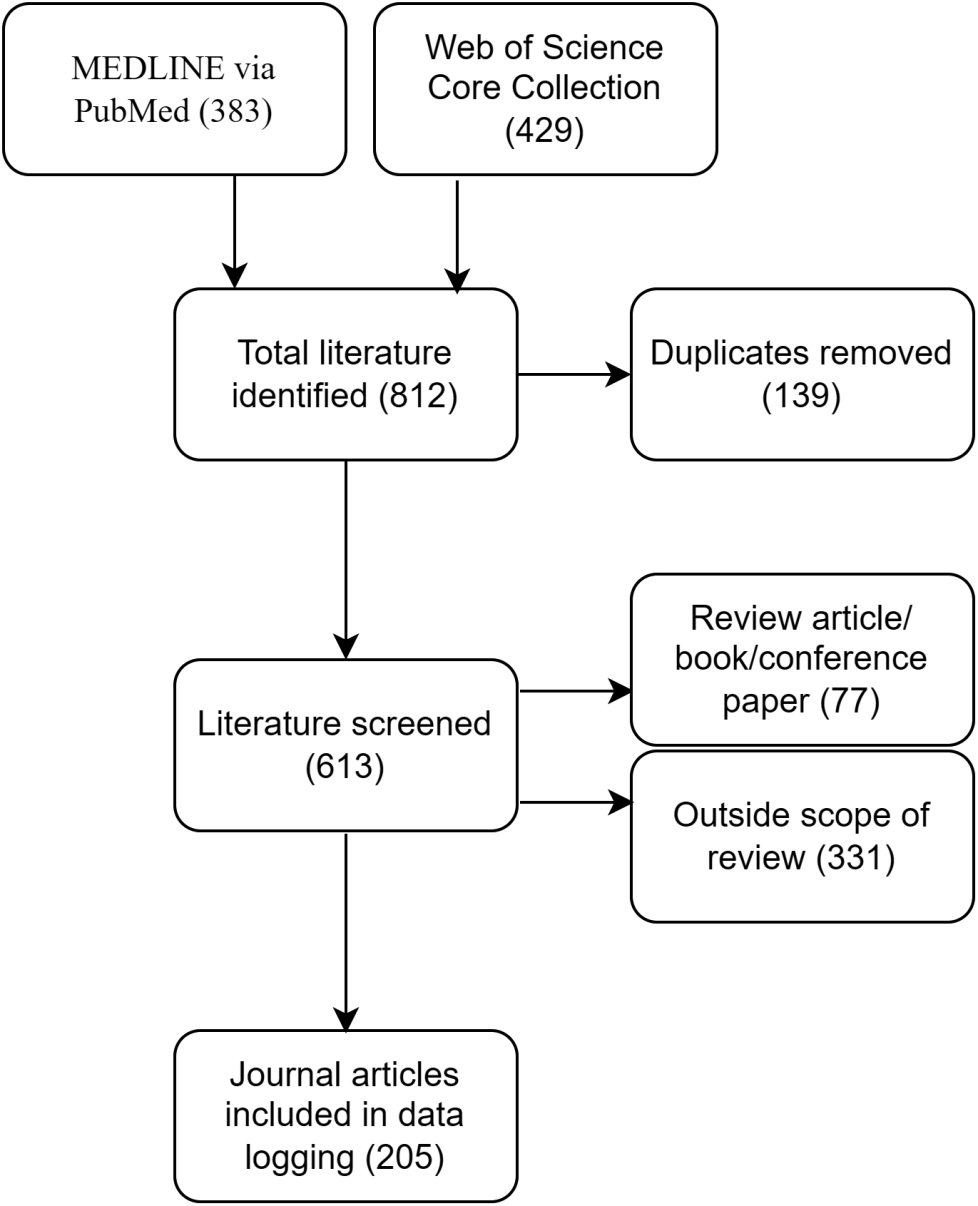
Flow chart illustrating the literature screening process (https://app.diagrams.net/). The number in parentheses indicates the number of articles at the given step.

MEDLINE via PubMed was searched on 1 June 2022 and yielded 383 citations (Fig. 1). Web of Science Core Collection was searched on 2 June 2022 and yielded 429 citations. After duplicates were removed, 613 citations remained. After removing review articles, books, and conference papers, we had 536 journal articles for relevance screening. Out of these articles, 331 were outside the scope of the review. Thus, a total of 205 journal articles were logged on the spreadsheet and included in this review (see Supplementary Text S1 for the full list of included journal articles).

**Fig. 2.**
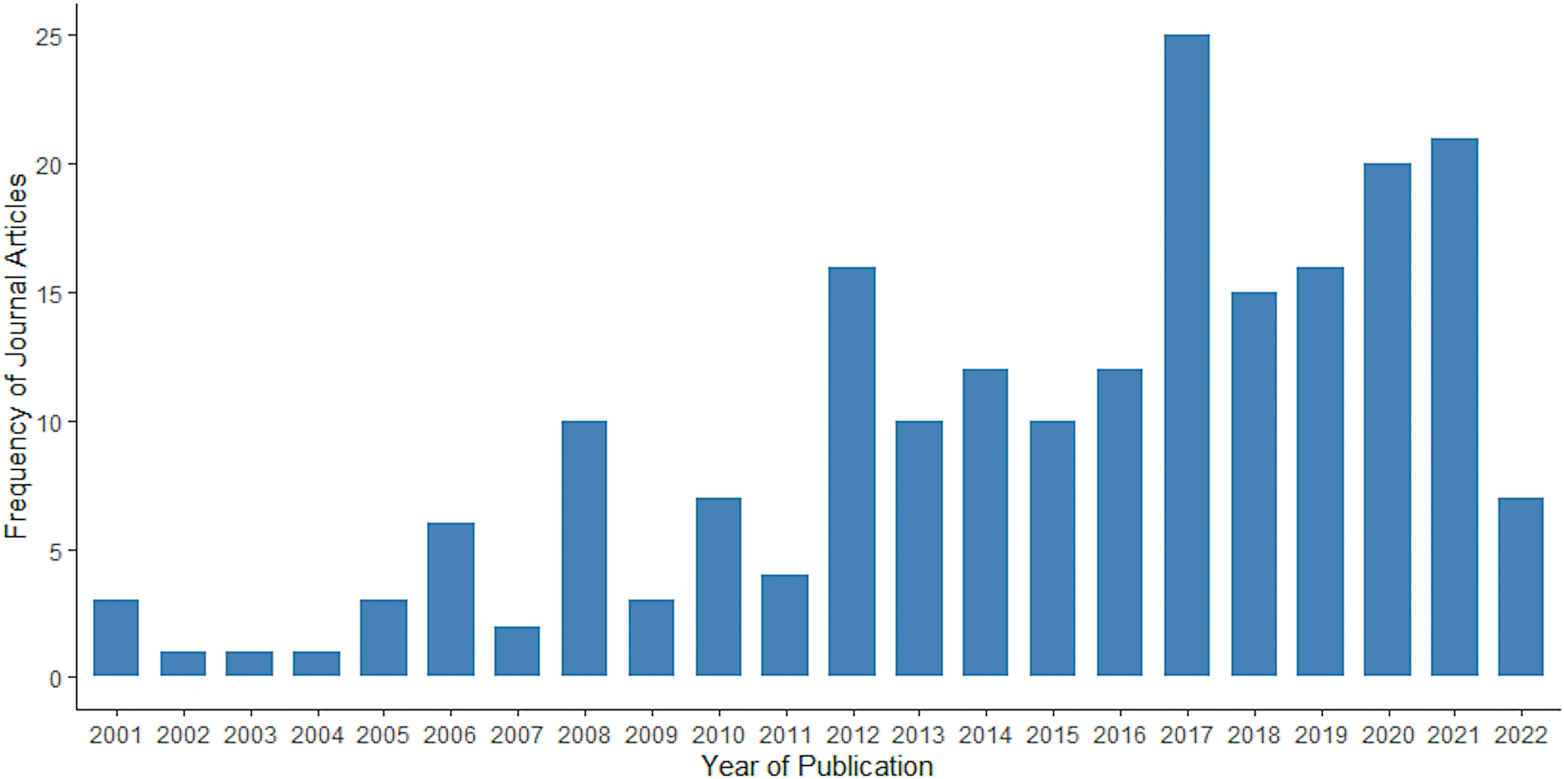
Frequency of the 205 included journal articles by year of publication.

The number of peer-reviewed journal articles that used environmental data to model vector-borne disease distributions and patterns increased over time, with the largest number of journal articles published in 2017 (Fig. 2). No journal articles that met the inclusion criteria for this review were published in 2000. From 2001 to 2010, only 37 relevant journal articles were published.

The most investigated diseases included West Nile virus infection (27.8%, *n* = 57), Lyme disease (20.5%, *n* = 42), and dengue fever (9.3%, *n* = 19) (Table 2). There were ≤5 journal articles per disease for the other vector-borne diseases that were investigated. Approximately 22.4% (*n* = 46) of the journal articles did not specify a vector-borne disease in their study (considered “Not specified” under “Disease” in Table 2), meaning that they investigated a vector or host/sentinel population as an indicator of human disease risk. Mosquitoes (56.6%, *n* = 116) and ticks (36.6%, *n* = 75) were the most investigated vectors.

**Table 2.** Study characteristics of the 205 included journal articles

Characteristic	Frequency	Percentage (%)
**Disease**		
Anaplasmosis	2	1.0
Anaplasmosis and ehrlichiosis	1	0.5
Babesiosis	1	0.5
Chagas disease	4	2.0
Chikungunya	1	0.5
Chikungunya, dengue fever, Zika	3	1.5
Dengue fever	19	9.3
Eastern equine encephalitis	4	2.0
Ehrlichiosis	3	1.5
Jamestown Canyon virus infection	1	0.5
La Crosse virus infection	1	0.5
Leishmaniasis	3	1.5
Lyme disease	42	20.5
Plague	5	2.4
Plague and tularemia	1	0.5
Rift Valley fever	1	0.5
Spotted fever group rickettsioses	4	2.0
St. Louis encephalitis virus infection	1	0.5
Tick-borne relapsing fever	1	0.5
West Nile virus infection	57	27.8
Zika	4	2.0
Not specified	46	22.4
**Vector**		
Flea	6	2.9
Flea and tick	1	0.5
Phlebotomine sandfly	3	1.5
Mosquito	116	56.6
Tick	75	36.6
Triatomine bug	4	2.0
**Geographic unit** ^a^		
City	14	6.8
County	20	9.8
State/province	69	33.7
US territory	4	2.0
Region	45	22.0
Country	37	18.0
North America	16	7.8
**Country**		
United States	150	73.2
Canada	21	10.2
Mexico	18	8.8

^a^A single journal article could include comparisons between different cities, counties, states, and regions. The only exception is country: if more than 1 country was included in the study, then “North America” was used as the geographic unit category.

The largest proportion of studies occurred at the state/province level (33.7%, *n* = 69) (Table 2). Puerto Rico was the only United States territory for which relevant journal articles were identified (2.0%, *n* = 4). Most studies investigated vector and vector-borne disease distribution in the United States (73.2%, *n* = 150) rather than in Canada, Mexico, or North America as a whole.

A wide range of modeling approaches for different populations of interest were utilized across the included literature. Regression was the most common type of modeling performed (41.0%, *n* = 84) (Table 3). Ecological niche modeling/habitat suitability was the next most prevalent model category (18.5%, *n* = 38), followed by compartment/deterministic/differential equations (11.2%, *n* = 23). Approximately 3.4% (*n* = 7) of the relevant publications employed models that fell into multiple categories and were equally important in the forecast determinations. The most commonly modeled population was vectors (67.8%, *n* = 139), followed by humans (40.0%, *n* = 82) and hosts/sentinels (20.4%, *n* = 42). Response variables for modeled populations included abundance (vectors), suitability/probability of presence of disease (vectors, humans), infection rates (vectors, host/sentinels), case counts (host/sentinels, humans), and case incidence (humans). One journal article (0.5%) served to compare models from other publications and did not produce a model of its own.

**Table 3.** Model characteristics of the 205 relevant journal articles

Characteristic	Frequency	Percentage (%)
**Primary model category**		
Compartmental/deterministic/differential equations	23	11.2
Ecological niche modeling/habitat suitability	38	18.5
Individual/agent-based	3	1.5
Machine learning	8	3.9
Multiple models^a^	7	3.4
Network analysis	1	0.5
Qualitative	1	0.5
Regression	84	41.0
Risk assessment metrics/correlations/empirical models	12	5.9
Ross-McDonald/*R*_0_/vectorial capacity	5	2.4
Spatial analyses	3	1.5
Spatiotemporal analyses	14	6.8
Survival analysis	1	0.5
Time series	5	2.4
**Population modeled** ^b^		
Reservoir host/sentinel	42	20.4
Human	82	40.0
Vector	139	67.8
Not applicable (model comparison)	1	0.5
**Environmental data** ^c^		
Elevation	49	23.9
Future climate scenarios/simulations	45	22.0
Meteorologic^d^	189	92.2
Satellite	58	28.3
Topography^e^	105	51.2
**Additional characteristics**		
Daylight	8	3.9
Drought	8	3.9
ENSO^f^	2	1.0
Experimental conditions	6	2.9
Fieldwork	12	5.9
Hurricane	2	1.0
Oceanographic^g^	1	0.5
Peer-reviewed literature	17	8.3

^a^Multiple kinds of models were used to address the aims of the study. Generally, different models were employed if the study had multiple aims.

^b^A single journal article may have collected data on multiple populations.

^c^A single journal article may have utilized multiple data sources. See Supplementary Text S2 for a full list of databases utilized.

^d^Meteorologic variables included temperature, growing degree days, precipitation, humidity, vapor pressure, and dew point.

^e^Topographic variables included land cover, hydrography, forest cover, soil type/texture, wetland cover, and stream data.

^f^ENSO index.

^g^Oceanographic variables refer to sea surface temperature.

To be included in the scoping review, analysis of environmental data was required. Environmental data sources and types were compiled from all 205 relevant journal articles. Most studies used data from the sources listed in Supplementary Text S2. Few studies pulled data from experimental environmental conditions (2.9%, *n* = 6), field work (5.9%, *n* = 12), or previously published data in other peer-reviewed literature (8.3%, *n* = 17) (Table 3). Meteorologic data were the most common type of environmental data to be included in a publication (92.2%, *n* = 189), followed by topographic data (51.2%, *n* = 105). Meteorological data was obtained from local observations (e.g., weather stations or field observations) to national or global gridded datasets of modeled or interpolated data (e.g., Daymet and WorldClim). Satellite data were utilized by 28.3% of relevant studies (*n* = 58), while future climate scenarios/simulations were utilized by 22.0% of relevant studies (*n* = 45). Additional environmental data types that few studies included were daylight (3.9%, *n* = 8), drought (3.9%, *n* = 8), the El Niño–Southern Oscillation (1.0%, *n* = 2), hurricanes (1.0%, *n* = 2), and oceanographic characteristics (0.5%, *n* = 1).

## Discussion and Conclusion

### Characteristics of Included Publications

The 3 most investigated diseases in the relevant literature were West Nile virus infection, Lyme disease, and dengue. This aligns with the most investigated vectors, which were mosquitoes (vectors of West Nile virus infection and dengue) and ticks (vectors of Lyme disease). All 3 of these diseases were among the top ten notifiable vector-borne diseases in the United States and associated territories in 2019. However, the frequency of vector-borne diseases by publication in this scoping review did not match the order of notifiable disease case numbers in the United States. In 2019, Lyme disease ranked first (39,945 cases), dengue ranked 7th (1,414 cases), and West Nile virus infection ranked 8th (974 cases) (Centers for Disease Control and Prevention 2019). This may reflect a tendency for researchers to focus on diseases of international importance and attention; the World Health Organization identifies dengue as 1 of the 5 major global vector-borne diseases, while West Nile virus infection and Lyme disease are recognized as vector-borne diseases of regional importance (World Health Organization 2017). Diseases of high priority tend to have more funded research and, thus, more available data, allowing for accurate model generation and parameterization. Despite the fact that *Anaplasma phagocytophilum* infection ranked second among the top 10 notifiable vector-borne diseases in the United States in 2019 (5,655 cases; Centers for Disease Control and Prevention 2019), it has a less well-known global presence and perhaps less available data. This may account for the presence of only 3 anaplasmosis publications in the relevant literature.

The 2 most studied geographic units were the state/province level and the region level, accounting for almost 56% of the included literature. This could be due to the ease and/or feasibility of using state/province or regional health department data, which are sometimes publicly available. However, access to health-related data can often be challenging due to privacy concerns. Additionally, models may fit better at the state/province or regional level due to fewer zeros in the outcome data than often occur at the smaller city or county scales. Only 25.8% of the included literature focused their analyses on country and continent (North America) geographic units. This suggests that it may not be as relevant to conduct vector-borne disease prediction at the country or continent level due to regional weather and climate variations, as well as disease and vector diversity. Access as well as quality and completeness of data aggregated across different administrative levels (e.g., states, counties, health departments) could also limit modeling at this spatial scale. Additionally, predictions at these coarse scales may be uninformative for use in vector control or public health decisions, which are necessarily very local in scope (Keyel et al. 2021).

### Use of Climate Data

While most studies included meteorological data in the analyses, these datasets vary in spatial and temporal resolution, potentially leading to variation in the generalizability of their results. Studies that gathered data from ground-based sources such as local weather stations likely had high accuracy but could be limited in generalizing results across space and time due to the limited number and placement of weather stations (Mendelsohn et al. 2007, Hooker et al. 2018). The incorporation of satellite data and gridded data products from organizations such as the National Aeronautics and Space Administration or the National Oceanic and Atmospheric Administration results in complete spatial coverage of chosen parameters over an environment but may not accurately reflect surface-level conditions (Mendelsohn et al. 2007).

All studies that pulled data from already existing sources were advantageous in that they used readily available and accessible data that future studies could reproduce. However, locally collected data through fieldwork focused on the location for which the author(s) built the model; data from other sources may not accurately represent the specific environment modeled. A future direction could include an assessment if the public health conclusion of a model meaningfully vary due to the type or source of climatic data included (e.g., local observations, remote sensed data, or interpolated data).

Another future direction could be a systematic assessment of the relative importance of climatic variables for the prediction or description of vector-borne diseases. These covariates are often included in models, given that vectors are known to be environmentally sensitive. However, the evaluation of the importance of climate covariates is not systematically performed. An evaluation of forecasts of dengue cases found that models that included climatic covariates performed worse than models that did not include climatic covariates (Johansson et al. 2019). However, in a similar multi-model assessment of forecasts of West Nile virus neuroinvasive disease cases, modeling frameworks that included climatic covariates performed, on average, better than those that did not (Holcomb et al. 2023a). However, no model outperformed a “simple” model using just historical case distributions (Holcomb et al. 2023a, 2023b).

### Gaps in Knowledge

Few journal articles identified in this scoping review focused their modeling efforts on anaplasmosis, spotted fever rickettsioses, babesiosis, ehrlichiosis, tularemia, or chikungunya. These diseases were among the top 10 notifiable vector-borne diseases in the United States and associated territories in 2019 (Centers for Disease Control and Prevention 2019). Future work should focus on these less commonly studied but highly impactful vector-borne diseases in North America.

A majority of the identified journal articles focused their modeling efforts on prediction in the United States; not many studies were conducted in Canada or Mexico. This could be due to resource and funding differences that lead to the availability of and variability in disease surveillance data and prediction activities (Connelly 2019, de Arias et al. 2022); attitudes and practices surrounding vector-borne disease control could vary by location, lowering researchers’ priority to conduct studies (Rosencrans et al. 2014, Aenishaenslin et al. 2016, Nava-Doctor et al. 2021); and publishing in a peer-reviewed journal could be difficult due to structural inequalities and lack of access in some areas (Man et al. 2004, Ekdale et al. 2022). These gaps and differences are concerning, due to documented range expansion of vectors, especially northward into Canada (Sonenshine 2018).

Animal reservoirs and sentinels were the least commonly modeled populations among the identified relevant journal articles. However, there are a number of studies and reviews that identify the value of using these groups as proxies for the human risk of disease (Kwan et al. 2010, Bowser and Anderson 2018, Clarke et al. 2022). As zoonotic disease reservoir populations change due to the changing climate, changes in vector-borne disease patterns in reservoirs could be used as early warning systems for humans.

### Strengths and Limitations

A similar scoping review had been performed to identify modeling efforts related to vector-borne diseases, using literature between 1999 and 2016 (Sadeghieh et al. 2020). However, we are not aware of a more recent scoping review nor any that specifically focuses on the use of climate data as it relates to vector-borne disease prediction in North America; the previous review focused on comparing modeling frameworks applied to locations around the globe. Therefore, this review adds to the body of literature by providing a 20-year overview of the state of North American vector-borne disease forecast research using climate data along with a catalog of data sources. Researchers can use this review as the basis for further critical assessments, including how different environmental data sources impact modeled relationships and the predictive power of environmental variables for predicting VBDs.

Limiting the eligibility criteria to articles published in English may have introduced language bias towards the United States. We may have identified more studies conducted in Mexico and Canada if we had included articles written in Spanish and French in our review.

By conducting the search using 2 relevant databases, it is likely that the journal articles included in this review provide a comprehensive representation of published literature on this topic. However, our selected keywords may not have captured all the relevant journal articles in these databases. In addition, we did not screen the reference lists of included publications. There is also the possibility that relevant journal articles were missed due to oversight during the literature screening process. Therefore, some relevant publications may have been missed in this review.

This review can serve as a resource for investigators incorporating environmental data into VBD forecasting and modeling efforts. They can identify published studies with similar aims and which study designs, environmental data sources, and modeling techniques have been incorporated in the past. Identifying and locating relevant environmental data sources can be time-consuming; thus, the catalog of data sources used in relatively recent publications can help to streamline the process. Further work needs to be done to assess if the use of different sources of the same environmental variable (e.g., temperature from gridded interpolated data or weather stations) meaningfully changes the public health interpretation of models and forecasts.

Future steps could also include critical evaluations and comparisons of VBD models. These in-depth comparisons were out of the scope of this review as our focus was on cataloguing the sources of climatic data incorporated in VBD models. Assessments of model accuracy, especially in relation to modeling framework and covariate inclusion, could identify useful techniques for future investigations. Similarly, a comparison of model diagnostics presented across models, as has been done previously (Sadeghieh et al. 2020), could be used to identify robust modeling frameworks or highlight areas needing further documentation in articles. Additionally, a comparison of model performance relative to meaningful baselines can identify relative improvements in different modeling frameworks, giving realistic assessments of model performances (Keyel and Kilpatrick 2023).

Researchers can also utilize this review to determine areas of future research interest. Based on the assessment conducted on the relevant literature, 4 avenues for future work come to light. First, additional studies are warranted to investigate lower incidences of VBDs as they have been conducted for West Nile virus infection, Lyme disease, and dengue. Second, future research should extend more broadly to geographic regions of North America outside of the United States. Third, researchers could explore less commonly used environmental variables. Some work in this area has been performed. Miley et al. (2020) examined the relationship between variable ENSO and eastern equine encephalitis in Florida, and Stapp et al. (2007) investigated the association between variable ENSO and plague in prairie dogs in Colorado. Laureano-Rosario et al. (2017) found that considering oceanographic characteristics, like sea surface temperature, improved the prediction of dengue fever for the northwest coast of Mexico. Additional research is warranted to ascertain how climatic conditions related to oceanographic conditions improve prediction and how these factors apply to inland mosquito populations. Finally, investigators should give attention to animal reservoir populations and sentinels as proxies for the risk of human disease.

## Supplementary Material

tjae031_suppl_Supplementary_Tables_S1

tjae031_suppl_Supplementary_Texts_S1

tjae031_suppl_Supplementary_Texts_S2
