# Supplementary material for: The use of environmental data in descriptive and predictive models of vector-borne disease in North America
Source: J Med Entomol. 2024 Mar 3;61(3):595–602. doi: 10.1093/jme/tjae031 (PMC11078578; doi:10.1093/jme/tjae031)
Supplement: tjae031_suppl_Supplementary_Texts_S1 [file tjae031_suppl_supplementary_texts_s1.docx]

**Supplemental Text S1. Full list of publications included in scoping review.**

**Alto BW, Juliano SA.** Precipitation and temperature effects on populations of *Aedes albopictus* (Diptera: Culicidae): implications for range expansion. *J Med Entomol.* **2001**:38(5):646–656. doi: 10.1603/0022-2585-38.5.646.

**Arora AK, Sim C, Severson DW, Kang DS.** Random forest analysis of impact of abiotic factors on *Culex pipiens* and *Culex quinquefasciatus* occurrence. *Front Ecol Evol.* **2022**:9:773360. doi: 10.3389/fevo.2021.773360.

**Baak-Baak CM, Moo-Llanes DA, Cigarroa-Toledo N, Puerto FI, Machain-Williams C, Reyes-Solis G, Nakazawa YJ, Ulloa-Garcia A, Garcia-Rejon JE.** Ecological niche model for predicting distribution of disease-vector mosquitoes in Yucatán State, México. *J Med Entomol.* **2017**:54(4):854–861. doi: 10.1093/jme/tjw243.

**Beeman SP, Morrison AM, Unnasch TR, Unnasch RS.** Ensemble ecological niche modeling of West Nile virus probability in Florida. *PLoS One.* **2021**:16(10):e0256868.

**Berger KA, Ginsberg HS, Dugas KD, Hamel LH, Mather TN.** Adverse moisture events predict seasonal abundance of Lyme disease vector ticks (*Ixodes scapularis*). *Parasit Vectors.* **2014**:7:181. doi: 10.1186/1756-3305-7-181.

**Boorgula CDY, Peterson AT, Foley DH, Ganta RR, Raghavan RK.** Assessing the current and future potential geographic distribution of the American dog tick, *Dermacentor variabilis* (Say) (Acari: Ixodidae) in North America. *PLoS One.* **2020**:15:e0237191. doi: 10.1371/journal.pone.0237191.

**Bouden M, Moulin B, Gosselin P.** The geosimulation of West Nile virus propagation: a multi-agent and climate sensitive tool for risk management in public health. *Int J Health Geogr.* **2008**:7:35. doi: 10.1186/1476-072X-7-35.

**Britch SC, Linthicum KJ, Anyamba A, Tucker CJ, Pak EW, Maloney FA, Cobb K, Stanwix E, Humphries J, Spring A, Pagac B, Miller M.** Satellite vegetation index data as a tool to forecast population dynamics of medically important mosquitoes at military installations in the continental United States. *Mil Med.* **2008:**173(7):677–683. doi: 10.7205/MILMED.173.7.677.

**Brown HE, Barrera R, Comrie AC, Lega J.** Effect of temperature thresholds on modeled *Aedes aegypti* (Diptera: Culicidae) population dynamics. *J Med Entomol.* **2017**:54(4): 869–877. doi: 10.1093/jme/tjx041.

**Brown HE, Cox J, Comrie AC, Barrera R.** Habitat and density of oviposition opportunity influences *Aedes aegypti* (Diptera: Culicidae) flight distance. *J Med Entomol.* 2017:54(5):1385–1389. doi: 10.1093/jme/tjx083.

**Brown HE, Ettestad P, Reynolds PJ, Brown TL, Hatton ES, Holmes JL, Glass GE, Gage KL, Eisen RJ.** Climatic predictors of the intra- and inter-annual distributions of plague cases in New Mexico based on 29 years of animal-based surveillance data. *Am J Trop Med Hyg.* **2010**:82(1):95–102. doi: 10.4269/ajtmh.2010.09-0247.

**Brown HE, Levy CE, Enscore RE, Schriefer ME, DeLiberto TJ, Gage KL, Eisen RJ.** Annual seroprevalence of *Yersinia pestis* in coyotes as predictors of interannual variation in reports of human plague cases in Arizona, United States. *Vector-Borne Zoonot.* **2011**:11(11):1439–1446. doi: 10.1089/vbz.2010.0196.

**Brown HE, Young A, Lega J, Andreadis TG, Schurich J, Comrie A.** Projection of climate change influences on US West Nile virus vectors. *Earth Interact.* **2015**:19:18. doi: 10.1175/EI-D-15-0008.1.

**Brownstein JS, Holford TR, Fish D.** A climate-based model predicts the spatial distribution of the Lyme disease vector *Ixodes scapularis* in the United States. *Environ Health Perspect.* **2003**:111(9):1152–1157. doi: 10.1289/ehp.6052.

**Brownstein JS, Holford TR, Fish D.** Effect of climate change on Lyme disease risk in North America. *Ecohealth.* **2005**:2(1):38–46. doi: 10.1007/s10393-004-0139-x.

**Burch C, Loraamm R, Unnasch T, Downs J.** Utilizing ecological niche modelling to predict habitat suitability of eastern equine encephalitis in Florida. *Ann GIS.* **2020**:26(2):133–147. doi: 10.1080/19475683.2020.1730962.

**Burkett-Cadena ND, Hassan HK, Eubanks MD, Cupp EW, Unnasch TR.** Winter severity predicts the timing of host shifts in the mosquito *Culex erraticus*. *Biol Lett.* **2012**:8(4):567–569. doi: 10.1098/rsbl.2012.0075.

**Bustamante DM, Lord CC.** Sources of error in the estimation of mosquito infection rates used to assess risk of arbovirus transmission. *Am J Trop Med Hyg.* **2010**:82(6):1172–1184. doi: 10.4269/ajtmh.2010.09-0323.

**Butterworth MK, Morin CW, Comrie AC.** An analysis of the potential impact of climate change on dengue transmission in the southeastern United States. *Environ Health Perspect.* **2017**:125(4):579–585. doi: 10.1289/EHP218.

**Byrd BD, Sither CB, Goggins JA, Kunze-Garcia S, Pesko KN, Bustamante DM, Sither JM, Vonesh JR, O’Meara GF.** Aquatic thermal conditions predict the presence of native and invasive rock pool *Aedes* (Diptera: Culicidae) in the southern Appalachians, U.S.A. *J Vector Ecol.* **2019**:44(1):30–39. doi: 10.1111/jvec.12326.

**Caillouët KA, Robertson SL.** Temporal and spatial impacts of hurricane damage on West Nile virus transmission and human risk. *J Am Mosq Control Assoc*. **2020**:36(2s):106–119. doi: 10.2987/19-6887.1.

**Cardenas R, Hussain-Alkhateeb L, Benitez-Valladares D, Sánchez-Tejeda G, Kroeger A.** The early warning and response system (EWARS-TDR) for dengue outbreaks: can it also be applied to chikungunya and Zika outbreak warning? *BMC Infect Dis.* **2022**:22:235. doi: 10.1186/s12879-022-07197-6.

**Carlson CJ, Bevins SN, Schmid BV.** Plague risk in the western United States over seven decades of environmental change. *Glob Change Biol.* **2022**:28(3):753–769. doi: 10.111/gcb.15966.

**Carmona-Castro O, Moo-Llanes DA, Ramsey JM.** Impact of climate change on vector transmission of *Trypanosoma cruzi* (Chagas, 1909) in North America. *Med Vet Entomol.* **2018**:32:84–101. doi: 10.1111/mve.12269.

**Castro LA, Fox SJ, Chen X, Liu K, Bellan SE, Dimitrov NB, Galvani AP, Meyers LA.** Assessing real-time Zika risk in the United States. *BMC Infect Dis.* **2017**:17:284. doi: 10.1186/s12879-017-2394-9.

**Chen CC, Jenkins E, Epp T, Waldner C, Curry PS, Soos C.** Climate change and West Nile virus in a highly endemic region of North America. *Int J Environ Res Public Health.* **2013**:10(7):3052–3071. doi: 10.3390/ijerph10073052.

**Chen CC, Epp T, Jenkins E, Waldner C, Curry PS, Soos C.** Predicting weekly variation of *Culex tarsalis* (Diptera: Culicidae) West Nile virus infection in a newly endemic region, the Canadian prairies. *J Med Entomol.* **2012**:49(5):1144–1153. doi: 10.1603.ME11221.

**Chen CC, Epp T, Jenkins E, Waldner C, Curry PS, Soos C.** Modeling monthly variation of *Culex tarsalis* (Diptera: Culicidae) abundance and West Nile virus infection rate in the Canadian prairies. *Int J Environ Res Public Health.* **2013**:10(7):3033–3051. doi: 10.3390/ijerph10073033.

**Chen D, Wong H, Belanger P, Moore K, Peterson M, Cunningham J.** Analyzing the correlation between deer habitat and the component of the risk for Lyme disease in eastern Ontario, Canada: a GIS-based approach. *ISPRS Int Geo-Inf.* **2015**:4(1):105–123. doi: 10.3390/ijgi4010105.

**Chuang T-W, Henebry GM, Kimball JS, Vanroekel-Patton DL, Hildreth MB, Wimberly MC.** Satellite microwave remote sensing for environmental modeling of mosquito population dynamics. *Remote Sens Environ.* **2012**:125:147–156. doi: 10.1016/j.rse.2012.07.018.

**Chuang TW, Wimberly MC.** Remote sensing of climatic anomalies and West Nile virus incidence in the northern Great Plains of the United States. *PLoS One.* **2012**:7(10):e46882. doi: 10.1371/journal.pone.0046882.

**Clarke-Crespo E, Moreno-Arzate CN, Lopez-Gonzalez CA.** Ecological niche models of four hard tick genera (Ixodidae) in Mexico. *Animals.* **2020**:10(4):649. doi: 10.3390/ani10040649.

**Cleckner HL, Allen TR, Bellows AS.** Remote sensing and modeling of mosquito abundance and habitats in coastal Virginia, USA. *Remote Sens.* **2011**:3(12):2663–2681. doi: 10.3390/rs3122663.

**Click Lambert R, Kolivras KN, Resler LM, Brewster CC, Paulson SL.** The potential for emergence of Chagas disease in the United States. *Geospat Health.* **2008**:2(2):227–239. doi: 10.4081/gh.2008.246.

**Clow KM, Ogden NH, Lindsay LR, Michel P, Pearl DL, Jardine CM.** The influence of abiotic and biotic factors on the invasion of *Ixodes scapularis* in Ontario, Canada. *Ticks Tick-Borne Dis.* **2017**:8(4):554–563. doi: 10.1016/j.ttbdis.2017.03.003.

**Colón-González FJ, Fezzi C, Lake IR, Hunter PR.** The effects of weather and climate change on dengue. *PLoS Negl Trop Dis.* **2013**:7(11):e2503. doi: 10.1371/journal.pntd.0002503.

**Cook C, Blesi A, Brozak S, Lenhart S, Reed H, Urquhart C, Moncayo A, Trout Fryxell R.** La Crosse virus spread within the mosquito population in Knox County, TN. *PLoS One.* **2021**:16(4):e0249811. doi: 10.1371/journal.pone.0249811.

**Couper L, MacDonald AJ, Mordecai EA.** Impact of prior and projected climate change on US Lyme disease incidence. *Glob Change Biol.* **2021**:27(4):738–754. doi: 10.1111/gcb.15435.

**Crowder DW, Dykstra EA, Brauner JM, Duffy A, Reed C, Martin E, Peterson W, Carrière Y, Dutilleul P, Owen JP.** West Nile virus prevalence across landscapes is mediated by local effects of agriculture on vector and host communities. *PLoS One.* **2013**:8(1):e55006. doi: 10.1371/journal.pone.0055006.

**Davis JK, Vincent GP, Hildreth MB, Kightlinger L, Carlson C, Wimberly MC.** Improving the prediction of arbovirus outbreaks: a comparison of climate-driven models for West Nile virus in an endemic region of the United States. *Acta Trop.* **2018**:185:242–250. doi: 10.1016/j.actatropica.2018.04.028.

**DeFelice NB, Birger R, DeFelice N, Gagner A, Campbell SR, Romano C, Santoriello M, Henke J, Wittie J, Cole B, Kaiser C, Shaman J.** Modeling and surveillance of reporting delays of mosquitoes and humans infected with West Nile virus and associations with accuracy of West Nile virus forecasts. *JAMA Netw Open.* **2019**:2(4):e193175. doi: 10.1001/jamanetworkopen.2019.3175.

**DeFelice NB, Schneider ZD, Little E, Barker C, Caillouet KA, Campbell SR, Damian D, Irwin P, Jones HMP, Townsend J, Shaman J.** Use of temperature to improve West Nile virus forecasts. *PLoS Comput Biol.* **2018**:14(3):e1006047. doi: 10.1371/journal.pcbi.1006047.

**DeGroote JP, Sugumaran R, Ecker M.** Landscape, demographic and climatic associations with human West Nile virus occurrence regionally in 2012 in the United States of America. *Geospat Health*. **2014**:9(1):153–168. doi: 10.4081/gh.2014.13.

**Dhingra R, Jimenez V, Chang HH, Gambhir M, Fu JS, Liu Y, Remais JV.** Spatially-explicit simulation modeling of ecological response to climate change: methodological considerations in predicting shifting population dynamics of infectious disease vectors. *ISPRS Int J Geo-Inf.* **2013**:2:645–664. doi: 10.3390/ijgi2030645.

**Diuk-Wasser MA, Brown HE, Andreadis TG, Fish D.** Modeling the spatial distribution of mosquito vectors for West Nile virus in Connecticut, USA. *Vector Borne Zoonotic Dis.* **2006**:6(3):283–295. doi: 10.1089/vbz.2006.6.283.

**Diuk-Wasser MA, Hoen AG, Cislo P, Brinkerhoff R, Hamer SA, Rowland M, Cortinas R, Vourc’h G, Melton F, Hickling GJ, Tsao JI, Bunikis J, Barbour AG, Kitron U, Piesman J, Fish D.** Human risk of infection with *Borrelia burgdorferi*, the Lyme disease agent, in eastern United States. *Am J Trop Med Hyg.* **2012**:86(2):320–327. doi: 10.4269.ajtmh.2012.11-0395.

**Diuk-Wasser MA, Vourc’h G, Cislo P, Hoen AG, Melton F, Hamer SA, Rowland M, Cortinas R, Hickling GJ, Tsao JI, Barbour AG, Kitron U, Piesman J, Fish D.** Field and climate-based model for predicting the density of host-seeking nymphal *Ixodes scapularis*, an important vector of tick-borne disease agents in the eastern United States. *Glob Ecol Biogeogr.* **2010**:19:504–514. doi: 10.1111/j.1466-8238.2010.00526.x.

**Donaldson TG, Perez de Leon AA, Li AI, Castro-Arellano I, Wozniak E, Boyle WK, Hargrove R, Wilder HK, Kim HJ, Teel PD, Lopez JE.** Assessment of the geographic distribution of *Ornithodoros turicata* (Argasidae): climate variation and host diversity. *Plos Neglect Trop Dis.* **2016**:10(3):e0004538. doi: 10.1371/journal.pntd.0004538.

**Dong Y, Huang Z, Zhang Y, Wang YXG, La Y.** Comparing the climatic and landscape risk factors for Lyme disease cases in the upper midwest and northeast United States. *Int J Environ Res Public Health.* **2020**:17(5):e1548. doi: 10.3390/ijerph17051548.

**Dumic I, Severnini E.** “Ticking bomb”: the impact of climate change on the incidence of Lyme disease. *Can J Infect Dis Med Microbiol.* **2018**:5719081. doi: 10.1155/2018/5719081.

**Eisen L, Eisen RJ, Lane RS.** Geographical distribution patterns and habitat suitability models for presence of host-seeking ixodid ticks in dense woodlands of Mendocino County, California. *J Med Entomol.* **2006**:43(2):415–427. doi: 10.1093/jmedent/43.2.415.

**Eisen L, Meyer AM, Eisen RJ.** Climate-based model predicting acarological risk of encountering the human-biting adult life stage of *Dermacentor andersoni* (Acari: Ixodidae) in a key habitat type in Colorado. *J Med Entomol.* **2007**:44(4):694–704. doi: 10.1093/jmedent/44.4.694.

**Eisen RJ, Clark RJ, Monaghan AJ, Eisen L, Delorey MJ, Beard CB.** Host-seeking phenology of *Ixodes pacificus* (Acari: Ixodidae) nymphs in northwestern California in relation to calendar week, woodland type, and weather conditions. *J Med Entomol.* **2017**:54(1):125–131. doi: 10.1093/jme/tjw155.

**Eisen RJ, Eisen L, Girard YA, Fedorova N, Mun J, Slikas B, Leonhard S, Kitron U, Lane RS.** A spatially-explicit model of acarological risk of exposure to *Borrelia burgdorferi*-infected *Ixodes pacificus* nymphs in northwestern California based on woodland type, temperature, and water vapor. *Ticks Tick-Borne Dis.* **2010**:1(1):35–43. doi: 10.1016/j.ttbdis.2009.12.002.

**Eisen RJ, Feirer S, Padgett KA, Hahn MB, Monaghan AJ, Kramer VL, Lane RS, Kelly M.** Modeling climate suitability of the western blacklegged tick in California. *J Med Entomol.* **2018**:55(5):1133–1142. doi: 10.1093/jme/tjy060.

**Elias SP, Gardner AM, Maasch KA, Birkel SD, Anderson NT, Rand PW, Lubelczyk CB, Smith RP.** A generalized additive model correlating blacklegged ticks with white-tailed deer density, temperature, and humidity in Maine, USA, 1990-2013. *J Med Entomol.* **2021**:58(1):125–138. doi: 10.1093/jme/tjaa180.

**Enscore RE, Biggerstaff BJ, Brown TL, Fulgham RE, Reynolds PJ, Engelthaler DM, Levy CE, Parmenter RR, Montenieri JA, Cheek JE, Grinnell RK, Ettestad PJ, Gage KL.** Modeling relationships between climate and the frequency of human plague cases in the southwestern United States, 1960-1997. *Am J Trop Med Hyg.* **2002**:66(2):186–196. doi: 10.4269/ajtmh.2002.66.186.

**Epp TY, Waldner CL, Berke O.** Predicting geographical human risk of West Nile virus - Saskatchewan, 2003 and 2007. *Can J Public Health.* **2009**:100(5):344–348. doi: 10.1007/BF03405266.

**Equihua M, Ibáñez-Bernal S, Benítez G, Estrada-Contreras I, Sandoval-Ruiz CA, Mendoza-Palmero FS.** Establishment of *Aedes aegypti* (L.) in mountainous regions in Mexico: increasing number of population at risk of mosquito-borne disease and future climate conditions. *Acta Trop.* **2017**:166:316–327. doi: 10.1016/j.actatropica.2016.11.014.

**Erickson RA, Hayhoe K, Presley SM, Allen LJS, Long KR, Cox SB.** Potential impacts of climate change on the ecology of dengue and its mosquito vector the Asian tiger mosquito (*Aedes albopictus*). *Environ Res Lett.* **2012**:7:034003. doi: 10.1088/1748-9326/7/3/034003.

**Evans MV, Hintz CW, Jones L, Shiau J, Solano N, Drake JM, Murdock CC.** Microclimate and larval habitat density predict adult *Aedes albopictus* abundance in urban areas. *Am J Trop Med Hyg.* **2019**:101(2):362–370. doi: 10.4269/ajtmh.19-0220.

**Fox SJ, Bellan SE, Perkins TA, Johansson MA, Meyers LA.** Downgrading disease transmission risk estimates using terminal importations. *Plos Neglect Trop Dis.* **2019**:13(6):e0007395. doi: 10.1371/journal.pntd.0007395.

**Gardner AM, Hamer GL, Hines AM, Newman CM, Walker ED, Ruiz MO.** Weather variability affects abundance of larval *Culex* (Diptera: Culicidae) in storm water catch basins in suburban Chicago. *J Med Entomol.* **2012**:49(2):270–276. doi: 10.1603/me11073.

**Gardner AM, Pawlikowski NC, Hamer SA, Hickling GJ, Miller JR, Schotthoefer AM, Tsao J, Allan BF.** Landscape features predict the current and forecast the future geographic spread of Lyme disease. *Proc R Soc B-Biol Sci.* **2020**:287:20202278. doi: 10.1098/rspb.2020.2278.

**Garza M, Arroyo TPF, Casillas EA, Sanchez-Cordero V, Rivaldi CL, Sarkar S.** Projected future distributions of vectors of *Trypanosoma cruzi* in North America under climate change scenarios. *Plos Neglect Trop Dis.* **2014**:8(5):e2818. doi: 10.1371/journal.pntd.0002818.

**Gatewood AG, Liebman KA, Vourc’h G, Bunikis J, Hamer SA, Cortinas R, Melton F, Cislo P, Kitron U, Tsao J, Barbour AG, Fish D, Diuk-Wasser MA.** Climate and tick seasonality are predictors of *Borrelia burgdorferi* genotype distribution. *Appl Environ Microbiol.* **2009**:75(8):2476–2483. doi: 10.1128/AEM.02633-08.

**Gibbs SEJ, Wimberly MC, Madden M, Masour J, Yabsley MJ, Stallknecht DE.** Factors affecting the geographic distribution of West Nile virus in Georgia, USA: 2002-2004. *Vector Borne Zoonotic Dis.* **2006**:6(1):73–82. doi: 10.1089/vbz.2006.6.73.

**Ginsberg HS, Albert M, Acevedo L, Dyer MC, Arsnoe IM, Tsao JI, Mather TN, LeBrun RA.** Environmental factors affecting survival of immature *Ixodes scapularis* and implications for geographical distribution of Lyme disease: the climate/behavior hypothesis. *PLoS One.* **2017**:12(1):e0168723. doi: 10.1371/journal/pone.0168723.

**Ginsberg HS, Rulison EL, Miller JL, Pang G, Arsnoe IM, Hickling GJ, Ogden NH, LeBrun RA, Tsao JI.** Local abundance of *Ixodes scapularis* in forests: effects of environmental moisture, vegetation characteristics, and host abundance. *Ticks Tick-Borne Dis.* **2020**:11(1):101271. doi: 10.1016/j.ttbdis.2019.101271.

**Giordano BV, Kaur S, Hunter FF.** West Nile virus in Ontario, Canada: a twelve-year analysis of human case prevalence, mosquito surveillance, and climate data. *PLoS One.* **2017**:12(8):e0183568. doi: 10.1371/journal.pone.0183568.

**Gluskin RT, Johansson MA, Santillana M, Brownstein JS.** Evaluation of internet-based dengue query data: Google dengue trends. *PLoS Negl Trop Dis.* **2014**:8(2):e2713. doi: 10.1371/journal.pntd.0002713.

**Gonzalez C, Rebollar-Tellez EA, Ibanez-Bernal S, Becker-Fauser I, Martinez-Meyer E, Townsend Peterson A, Sanchez-Cordero V.** Current knowledge of leishmania vectors in Mexico: how geographic distributions of species relate to transmission areas. *Am J Trop Med Hyg.* **2011**:85(5):839–846. doi: 10.4269/ajtmh.2011.10-0452.

**González C, Wang O, Strutz SE, González-Salazar C, Sánchez-Cordero V, Sarkar S.** Climate change and risk of leishmaniasis in North America: predictions from ecological niche models of vector and reservoir species. *PLoS Negl Trop Dis.* **2010:**4(1):e585. doi: 10.1371/journal.pntd.0000585.

**Hahn MB, Feirer S, Monaghan AJ, Lane RS, Eisen RJ, Padgett KA, Kelly M.** Modeling future climate suitability for the western blacklegged tick, *Ixodes pacificus*, in California with an emphasis on land access and ownership. *Ticks Tick-Borne Dis.* **2021**:12(5):101789. doi: 10.1016/j.ttbdis.2021.101789.

**Hahn MB, Jarnevich CS, Monaghan AJ, Eisen RJ.** Modeling the geographic distribution of *Ixodes scapularis* and *Ixodes pacificus* (Acari: Ixodidae) in the contiguous United States. *J Med Entomol.* **2016**:53(5):1176–1191. doi: 10.1093/jme/tjw076.

**Hahn MB, Monaghan AJ, Hayden MH, Eisen RJ, Delorey MJ, Lindsey NP, Nasci RS, Fischer M.** Meteorological conditions associated with increased incidence of West Nile virus disease in the United States, 2004-2012. *Am J Trop Med Hyg.* **2015**:92(5):1013–1022. doi: 10.4269/ajtmh.14-0737.

**Harrigan RJ, Thomassen HA, Buermann W, Smith TB.** A continental risk assessment of West Nile virus under climate change. *Glob Chang Biol.* **2014**:20(8): 2417–2425. doi: 10.1111/gcb.12534.

**Hartley DM, Barker CM, Le Menach A, Niu T, Gaff HD, Reisen WK.** Effects of temperature on emergence and seasonality of West Nile virus in California. *Am J Trop Med Hyg.* **2012**:86(5):884–894. doi: 10.4269/ajtmh.2012.11-0342.

**Hayes LE, Scott JA, Stafford KC.** Influences of weather on *Ixodes scapularis* nymphal densities at long-term study sites in Connecticut. *Ticks Tick-Borne Dis.* **2015**:6(3):258–266. doi: 10.1016/j.ttbdis.2015.01.006.

**Hess A, Davis JK, Wimberly MC.** Identifying environmental risk factors and mapping the distribution of West Nile virus in an endemic region of North America. *Geohealth.* **2018**:2(12):395–409. doi: 10.1029/2018GH000161.

**Hollis-Etter KM, Montgomery RA, Etter DR, Anchor CL, Chelsvig JE, Warner RE, Grimstad PR, Lovin DD, Godsey MS.** Environmental conditions for Jamestown Canyon virus correlated with population-level resource selection by white-tailed deer in a suburban landscape. *PLoS One.* **2019**:14(10):e0223582. doi: 10.1371/journal.pone.0223582.

**Hongoh V, Berrang-Ford L, Scott ME, Lindsay LR.** Expanding geographical distribution of the mosquito, *Culex pipiens*, in Canada under climate change. *Appl Geogr.* **2012**:33:53–62. doi: 10.1016/j.apgeog.2011.05.015.

**Humphreys JM, Pelzel-McCluskey AM, Cohnstaedt LW, McGregor BL, Hanley KA, Hudson AR, Young K, Peck D, Rodriguez LL, Peters DPC.** Integrating spatiotemporal epidemiology, eco-phylogenetics, and distributional ecology to assess West Nile disease risk in horses. *Viruses.* **2021**:13(9):1811. doi: 10.3390/v13091811.

**Humphreys JM, Young KI, Cohnstaedt LW, Hanley KA, Peters DPC.** Vector surveillance, host species richness, and demographic factors as West Nile disease risk indicators. *Viruses.* **2021**:13(5):934. doi: 10.3390/v13050934.

**Johansson MA, Dominici F, Glass GE.** Local and global effects of climate on dengue transmission in Puerto Rico. *PLoS Neglect Trop Dis.* **2009**:3(2):e382. doi: 10.1371/journal.pntd.0000382.

**Johansson MA, Reich NG, Hota A, Brownstein JS, Santillana M.** Evaluating the performance of infectious disease forecasts: a comparison of climate-driven and seasonal dengue forecasts for Mexico. *Sci Rep.* **2016**:6:33707. doi: 10.1038/srep33707.

**Johnson TL, Bjork JKH, Neitzel DF, Dorr FM, Schiffman EK, Eisen RJ.** Habitat suitability model for the distribution of *Ixodes scapularis* (Acari: Ixodidae) in Minnesota. *J Med Entomol.* **2016**:53(3):598–606. doi: 10.1093/jme/tjw008.

**Karki S, Brown WM, Uelmen J, Ruiz MO, Smith RL.** The drivers of West Nile virus human illness in the Chicago, Illinois, USA area: fine scale dynamic effects of weather, mosquito infection, social, and biological conditions. *PLoS One.* **2020**:15(5):e0227160. doi: 10.1371/journal.pone.0227160.

**Karki S, Westcott NE, Muturi EJ, Brown WM, Ruiz MO.** Assessing human risk of illness with West Nile virus mosquito surveillance data to improve public health preparedness. *Zoonoses Public Health.* **2018**:65(1):177–184. doi: 10.1111/zph.12386.

**Kerins JL, Dorevitch S, Dworkin MS.** Spotted fever group rickettsioses (SFGR): weather and incidence in Illinois. *Epidemiol Infect.* **2017**:145(12):2466–2472. doi: 10.1017/S0950268817001492.

**Kessler WH, Blackburn JK, Sayler KA, Glass GE.** Estimating the geographic distribution of host-seeking adult *Amblyomma americanum* (Acari: Ixodidae) in Florida. *J Med Entomol.* **2019**:56(1):55–64. doi: 10.1093/jme/tjy147.

**Keyel AC, Elison Timm O, Backenson PB, Prussing C, Quinones S, McDonough KA, Vuille M, Conn JE, Armstrong PM, Andreadis TG, Kramer LD.** Seasonal temperatures and hydrological conditions improve the prediction of West Nile virus infection rates in Culex mosquitoes and human case counts in New York and Connecticut. *PLoS One.* **2019**:14(6):e0217854. doi: 10.1371/journal.pone.0217854.

**Keyel AC, Raghavendra A, Ciota AT, Elison Timm O.** West Nile virus is predicted to be more geographically widespread in New York State and Connecticut under future climate change. *Glob Chang Biol.* **2021**:27(21):5430–5445. doi: 10.1111/gcb.15842.

**Khatchikian CE, Prusinski M, Stone M, Backenson PB, Wang IN, Levy MZ, Brisson D.** Geographical and environmental factors driving the increase in the Lyme disease vector *Ixodes scapularis*. *Ecosphere.* **2012**:3(10):art85. doi: 10.1890/ES12-00134.1.

**Kinney AC, Current S, Lega J.** Aedes-AI: neural network models of mosquito abundance. *PLoS Comput Biol.* **2021**:17(11):e1009467. doi: 10.1371/journal.pcbi.1009467.

**Konrad SK, Miller SN**. A temperature-limited assessment of the risk of Rift Valley fever transmission and establishment in the continental United States of America. *Geospat Health.* **2012**:6:161–170. doi: 10.4081/gh.2012.134.

**Kwan JL, Park BK, Carpenter TE, Ngo V, Civen R, Reisen WK.** Comparison of enzootic risk measures for predicting West Nile disease, Los Angeles, California, USA, 2004-2010. *Emerg Infect Dis.* **2012**:18:1298–1306. doi: 10.3201/eid1808.111558.

**LaDeau SL, Calder CA, Doran PJ, Marra PP.** West Nile virus impacts in American crow populations are associated with human land use and climate. *Ecol Res.* **2011**:26:909–916. doi: 10.1007/s11284-010-0725-z.

**Lantos PM, Tsao J, Janko M, Arab A, von Fricken ME, Auwaerter PG, Nigrovic LE, Fowler V, Ruffin F, Gaines D, Broyhill J, Swenson J.** Environmental correlates of Lyme disease emergence in southwest Virginia, 2005-2014. *J Med Entomol.* **2021**:58(4):1680–1685. doi: 10.1093/jme/tjab038.

**Laureano-Rosario AE, Garcia-Rejon JE, Gomez-Carro S, Farfan-Ale JA, Muller-Karger FE.** Modelling dengue fever risk in the State of Yucatan, Mexico using regional-scale satellite-derived sea surface temperature. *Acta Trop*. **2017**:172:50–57. doi: 10.1016/j.actatropica.2017.04.017.

**Leighton PA, Koffi JK, Pelcat Y, Lindsay LR, Ogden NH.** Predicting the speed of tick invasion: an empirical model of range expansion for the Lyme disease vector *Ixodes scapularis* in Canada. *J Appl Ecol*. **2012**:49(2):457–464. doi: 10.1111/j.1365-2664.2012.02112.x.

**Lieske DJ, Lloyd VK.** Combining public participatory surveillance and occupancy modelling to predict the distributional response of *Ixodes scapularis* to climate change. Ticks Tick Borne Dis. **2018**:9(3):695–706. doi: 10.1016/j.ttbdis.2018.01.018.

**Lin S, Shrestha S, Prusinski MA, White JL, Lukacik G, Smith M, Lu J, Backenson B.** The effects of multiyear and seasonal weather factors on incidence of Lyme disease and its vector in New York State. *Sci Total Environ*. **2019**:665:1182–1188. doi: 10.1016/j.scitotenv.2019.02.123.

**Little E, Biehler D, Leisnham PT, Jordan R, Wilson S, LaDeau SL.** Socio-ecological mechanisms supporting high densities of *Aedes albopictus* (Diptera: Culicidae) in Baltimore, MD. *J Med Entomol*. **2017**:54(5):1183–1192. doi: 10.1093/jme/tjx103.

**Little E, Campbell SR, Shaman J.** Development and validation of a climate-based ensemble prediction model for West Nile Virus infection rates in *Culex* mosquitoes, Suffolk County, New York. *Parasit Vectors*. **2016**:9:443. doi: 10.1186/s13071-016-1720-1.

**Liu Y, Hu J, Snell-Feikema I, VanBemmel MS, Lamsal A, Wimberly MC.** Software to facilitate remote sensing data access for disease early warning systems. *Environ Modell Softw.* **2015**:74:247–257. doi: 10.1016/j.envsoft.2015.07.006.

**Liu Y, Lund RB, Nordone SK, Yabsley MJ, McMahan CS.** A Bayesian spatio-temporal model for forecasting the prevalence of antibodies to *Ehrlichia* species in domestic dogs within the contiguous United States. Parasit Vectors. **2017**:10:138. doi: 10.1186/s13071-017-2068-x.

**Liu Y, Watson SC, Gettings JR, Lund RB, Nordone SK, Yabsley MJ, McMahan CS.** A Bayesian spatio-temporal model for forecasting *Anaplasma* species seroprevalence in domestic dogs within the contiguous United States. *PLoS One.* **2017**:12(7):e0182028. doi: 10.1371/journal.pone.0182028.

**Lopez-Cardenas J, Bravo FEG, Schettino PMS, Solorzano JCG, Barba ER, Mendez JM, Sanchez-Cordero V, Peterson AT, Ramsey JM.** Fine-scale predictions of distributions of Chagas disease vectors in the state of Guanajuato, Mexico. *J Med Entomol*. **2005**:42(6):1068–1081. doi: 10.1093/jmedent/42.6.1068.

**López-Pérez AM, Gage K, Rubio AV, Montenieri J, Orozco L, Suzan G.** Drivers of flea (Siphonaptera) community structure in sympatric wild carnivores in northwestern Mexico. *J Vector Ecol*. **2018**:43(1):15–25. doi: 10.1111/jvec.12278.

**Lord CC, Day JF.** Simulation studies of St. Louis encephalitis virus in south Florida. *Vector Borne Zoonotic Dis*. **2001**:1(4):299–315. doi: 10.1089/15303660160025921.

**Lorenz A, Dhingra R, Chang HH, Bisanzio D, Liu Y, Remais JV.** Inter-model comparison of the landscape determinants of vector-borne disease: implications for epidemiological and entomological risk modeling. *PLoS One*. **2014**:9(7):e103163. doi: 10.1371/journal.pone.0103163.

**Ludwig A, Ginsberg HS, Hickling GJ, Ogden NH**. A dynamic population model to investigate effects of climate and climate-independent factors on the lifecycle of *Amblyomma americanum* (Acari: Ixodidae). *J Med Entomol*. **2016**:53(1):99–115. doi: 10.1093/jme/tjv150.

**Ma Y, He G, Yang R, Wang YXG, Huang ZYX, Dong Y.** Effect of land-use change on the changes in human Lyme risk in the United States. *Sustainability*. **2022**:14(10):5802. doi: 10.3390/su14105802.

**MacDonald AJ.** Abiotic and habitat drivers of tick vector abundance, diversity, phenology, and human encounter risk in southern California. *PLoS One*. **2018**:13(7):e0201665. doi: 10.1371/journal.pone.0201665.

**MacDonald AJ, Hyon DW, Brewington JB, O’Connor KE, Swei A, Briggs CJ.** Lyme disease risk in southern California: abiotic and environmental drivers of *Ixodes pacificus* (Acari: Ixodidae) density and infection prevalence with *Borrelia burgdorferi*. *Parasit Vectors*. **2017**:10:7. doi: 10.1186/s13071-016-1938-y.

**MacDonald AJ, McComb S, O’Neill C, Padgett KA, Larsen AE.** Projected climate and land use change alter western blacklegged tick phenology, seasonal host-seeking suitability and human encounter risk in California. *Glob Chang Biol.* **2020**:26(10):5459–5474. doi: 10.1111/gcb.15269.

**Mallya S, Sander B, Roy-Gagnon MH, Taljaard M, Jolly A, Kulkarni MA.** Factors associated with human West Nile virus infection in Ontario: a generalized linear mixed modelling approach. *BMC Infect Dis*. **2018**:18:141. doi: 10.1186/s12879-018-3052-6.

**Manore CA, Davis JK, Christofferson RC, Wesson DM, Hyman JM, Mores CN.** Towards an early warning system for forecasting human West Nile virus incidence. *PLoS Curr*. **2014**:6:ecurrents.outbreaks.f0b3978230599a56830ce30cb9ce0500. doi: 10.1371/currents.outbreaks.ed6f0f8a61d20ae5f32aaa5c2b8d3c23.

**Martínez-Vega RA, Danis-Lozano R, Velasco-Hernández J, Díaz-Quijano FA, González-Fernández M, Santos R, Román S, Argáez-Sosa J, Nakamura M, Ramos-Castañeda J.** A prospective cohort study to evaluate peridomestic infection as a determinant of dengue transmission: protocol. *BMC Public Health.* **2012**:12:262. doi: 10.1186/1471-2458-12-262.

**McClure M, Diuk-Wasser MA.** Climate impacts on blacklegged tick host-seeking behavior. *Int J Parasit.* **2019**:49(1):37–47. doi: 10.1016/j.ijpara.2018.08.005.

**McMahan CS, Wang D, Beall MJ, Bowman DD, Little SE, Pithua PO, Sharp JL, Stich RW, Yabsley MJ, Lund RB.** Factors associated with *Anaplasma* spp. seroprevalence among dogs in the United States. *Parasit Vectors*. **2016**:9:169. doi: 10.1186/s13071-016-1431-7.

**McPherson M, García-García A, Cuesta-Valero FJ, Beltrami H, Hansen-Ketchum P, MacDougall D, Ogden NH.** Expansion of the Lyme disease vector *Ixodes scapularis* in Canada inferred from CMIP5 climate projections. *Environ Health Perspect*. **2017**:125(5):057008. doi: 10.1289/ehp57.

**Miley KM, Downs J, Beeman SP, Unnasch TR.** Impact of the Southern Oscillation Index, temperature, and precipitation on eastern equine encephalitis virus activity in Florida. *J Med Entomol.* **2020**:57(5):1604–1613. doi: 10.1093/jme/tjaa084.

**Minigan JN, Hager HA, Peregrine AS, Newman JA.** Current and potential future distribution of the American dog tick (*Dermacentor variabilis*, Say) in North America. *Ticks Tick Borne Dis*. **2018**:9(2):354–362. doi: 10.1016/j.ttbdis.2017.11.012.

**Monaghan AJ, Moore SM, Sampson KM, Beard CB, Eisen RJ.** Climate change influences on the annual onset of Lyme disease in the United States. *Ticks Tick Borne Dis.* **2015**:6(5):615–622. doi: 10.1016/j.ttbdis.2015.05.005.

**Moo-Llanes DA, de Oca-Aguilar ACM, Romero-Salas D, Sanchez-Montes S.** Inferring the potential distribution of an emerging rickettsiosis in America: the case of *Rickettsia parkeri*. *Pathogens*. **2021**:10(5):592. doi: 10.3390/pathogens10050592.

**Moo-Llanes D, Ibarra-Cerdeña CN, Rebollar-Téllez EA, Ibáñez-Bernal S, González C, Ramsey JM.** Current and future niche of North and Central American sand flies (Diptera: Psychodidae) in climate change scenarios. *PLoS Negl Trop Dis*. **2013**:7(9):e2421. doi: 10.1371/journal.pntd.0002421.

**Moore SM, Eisen RJ, Monaghan A, Mead P.** Meteorological influences on the seasonality of Lyme disease in the United States. *Am J Trop Med Hyg*. **2014**:90(3):486–496. doi: 10.4269/ajtmh.13-0180.

**Moreno-Madrinan MJ, Crosson WL, Eisen L, Estes SM, Estes MG, Hayden M, Hemmings SN, Irwin DE, Lozano-Fuentes S, Monaghan AJ, Quattrochi D, Welsh-Rodriguez CM, Zielinski-Gutierrez E.** Correlating remote sensing data with the abundance of pupae of the dengue virus mosquito vector, *Aedes aegypti*, in Central Mexico. *ISPRS Int Geo-Inf.* **2014**:3(2):732–749. doi: 10.3390/ijgi3020732.

**Morin CW, Comrie AC.** Modeled response of the West Nile virus vector *Culex quinquefasciatus* to changing climate using the dynamic mosquito simulation model. *Int J Biometeorol*. **2010**:54(5):517–529. doi: 10.1007/s00484-010-0349-6.

**Morin CW, Comrie AC.** Regional and seasonal response of a West Nile virus vector to climate change. *Proc Natl Acad Sci USA*. **2013**:110(39):15620–15625. doi: 10.1073/pnas.1307135110.

**Morin CW, Monaghan AJ, Hayden MH, Barrera R, Ernst K.** Meteorologically driven simulations of dengue epidemics in San Juan, PR. *PLoS Negl Trop Dis*. **2015**:9(8):e0004002. doi: 10.1371/journal.pntd.0004002.

**Moua Y, Kotchi SO, Ludwig A, Brazeau S.** Mapping the habitat suitability of West Nile virus vectors in southern Quebec and eastern Ontario, Canada, with species distribution modeling and satellite Earth observation data. **Remote Sens**. **2021**:13(9):1637. doi: 10.3390/rs13091637.

**Mowry S, Keesing F, Fischhoff IR, Ostfeld RS.** Predicting larval tick burden on white-footed mice with an artificial neural network. *Ecol Inform.* **2019**:52:150–158. doi: 10.1016/j.ecoinf.2019.04.002.

**Muñoz ÁG, Chourio X, Rivière-Cinnamond A, Diuk-Wasser MA, Kache PA, Mordecai EA, Harrington L, Thomson MC.** AeDES: a next-generation monitoring and forecasting system for environmental suitability of *Aedes*-borne disease transmission. *Sci Rep*. **2020**:10(1):12640. doi: 10.1038/s41598-020-69625-4.

**Myer MH, Fizer CM, Mcpherson KR, Neale AC, Pilant AN, Rodriguez A, Whung PY, Johnston JM.** Mapping Aedes aegypti (Diptera: Culicidae) and *Aedes albopictus* vector mosquito distribution in Brownsville, TX. *J Med. Entomol.* **2020**:57(1):231–240. doi: 10.1093/jme/tjz132.

**Nakazawa Y, Williams R, Peterson AT, Mead P, Staples E, Gage KL.** Climate change effects on plague and tularemia in the United States. *Vector Borne Zoonotic Dis*. **2007**:7(4):529–540. doi: 10.1089/vbz.2007.0125.

**Nasrinpour HR, Reimer AA, Friesen MR, McLeod RD.** Data preparation for West Nile virus agent-based modelling: protocol for processing bird population estimates and incorporating ArcMap in AnyLogic. *JMIR Res Protoc*. **2017**:6(7):e138. doi: 10.2196/resprot.6213.

**Ng V, Fazil A, Gachon P, Deuymes G, Radojević M, Mascarenhas M, Garasia S, Johansson MA, Ogden NH.** Assessment of the probability of autochthonous transmission of chikungunya virus in Canada under recent and projected climate change. *Environ Health Perspect*. **2017**:125(5):067001. doi: 10.1289/ehp669.

**Ngonghala CN, Ryan SJ, Tesla B, Demakovsky LR, Mordecai EA, Murdock CC, Bonds MH.** Effects of changes in temperature on Zika dynamics and control. *J R Soc Interface*. **2021**:18(178):20210165. doi: 10.1098/rsif.2021.0165.

**Nielsen CF, Armijos MV, Wheeler S, Carpenter TE, Boyce WM, Kelley K, Brown D, Scott TW, Reisen WK.** Risk factors associated with human infection during the 2006 West Nile virus outbreak in Davis, a residential community in northern California. *Am J Trop Med Hyg*. **2008**:78(1):53–62. doi: 10.4269/ajtmh.2008.78.53.

**Nova N, Deyle ER, Shocket MS, MacDonald AJ, Childs ML, Rypdal M, Sugihara G, Mordecai EA.** Susceptible host availability modulates climate effects on dengue dynamics. *Ecol Lett*. **2021**:24(3):415–425. doi: 10.1111/ele.13652.

**Nunez-Lopez M, Ramos LA, Velasco-Hernandez JX.** Migration rate estimation in an epidemic network. *Appl Math Model*. **2021**:89:1949–1964. doi: 10.1016/j.apm.2020.08.025.

**Obenauer JF, Andrew Joyner T, Harris JB.** The importance of human population characteristics in modeling *Aedes aegypti* distributions and assessing risk of mosquito-borne infectious diseases. *Trop Med Health*. **2017**:45:38. doi: 10.1186/s41182-017-0078-1.

**Ogden NH, Maarouf A, Barker IK, Bigras-Poulin M, Lindsay LR, Morshed MG, O’callaghan CJ, Ramay F, Waltner-Toews D, Charron DF.** Climate change and the potential for range expansion of the Lyme disease vector *Ixodes scapularis* in Canada. *Int J Parasitol.* **2006**:36(1):63–70. doi: 10.1016/j.ijpara.2005.08.016.

**Ogden NH, Milka R, Caminade C, Gachon P.** Recent and projected future climatic suitability of North America for the Asian tiger mosquito *Aedes albopictus*. *Parasit Vectors*. **2014**:7:532. doi: 10.1186/s13071-014-0532-4.

**Ogden NH, Radojevic M, Wu X, Duvvuri VR, Leighton PA, Wu J.** Estimated effects of projected climate change on the basic reproductive number of the Lyme disease vector *Ixodes scapularis*. *Environ Health Perspect*. **2014**:122(6):631–638. doi: 10.1289/ehp.1307799.

**Ogden NH, St-Onge L, Barker IK, Brazeau S, Bigras-Poulin M, Charron DF, Francis CM, Heagy A, Lindsay LR, Maarouf A, Michel P, Milord F, O’Callaghan CJ, Trudel L, Thompson RA.** Risk maps for range expansion of the Lyme disease vector, *Ixodes scapularis*, in Canada now and with climate change. *Int J Health Geogr*. **2008**:7:24. doi: 10.1186/1476-072x-7-24.

**Okuneye KO, Velasco-Hernandez JX, Gumel AB.** The “unholy” chikungunya-dengue-Zika trinity: a theoretical analysis. *J Biol Syst*. **2017**:25(4):545–585. doi: 10.1142/S0218339017400046.

**Ostfeld RS, Canham CD, Oggenfuss K, Winchcombe RJ, Keesing F.** Climate, deer, rodents, and acorns as determinants of variation in Lyme-disease risk. *PLoS Biol.* **2006**:4(6):1058–1068. doi: 10.1371/journal.pbio.0040145.

**Ostfeld RS, Schauber EM, Canham CD, Keesing F, Jones CG, Wolff JO.** Effects of acorn production and mouse abundance on abundance and *Borrelia burgdorferi* infection prevalence of nymphal *Ixodes scapularis* ticks. *Vector Borne Zoonotic Dis*. **2001**:1(1):55–63. doi: 10.1089/153036601750137688.

**Peper ST, Dawson DE, Dacko N, Athanasiou K, Hunter J, Loko F, Almas S, Sorensen GE, Urban KN, Wilson-Fallon AN, Haydett KM, Greenberg HS, Gibson AG, Presley SM.** Predictive modeling for West Nile virus and mosquito surveillance in Lubbock, Texas. *J Am Mosq Control Assoc*. **2018**:34(1):18–24. doi: 10.2987/17-6714.1.

**Pepin KM, Eisen RJ, Mead PS, Piesman J, Fish D, Hoen AG, Barbour AG, Hamer S, Diuk-Wasser MA.** Geographic variation in the relationship between human Lyme disease incidence and density of infected host-seeking *Ixodes scapularis* nymphs in the eastern United States. *Am J Trop Med Hyg*. **2012**:86(6):1062–1071. doi: 10.4269/ajtmh.2012.11-0630.

**Peterson AT, Martinez-Campos C, Nakazawa Y, Martinez-Meyer E.** Time-specific ecological niche modeling predicts spatial dynamics of vector insects and human dengue cases. *Trans Roy Soc Trop Med Hyg*. **2005**:99(9):647–655. doi: 10.1016/j.trstmh.2005.02.004.

**Peterson AT, Robbins A, Restifo R, Howell J, Nasci R.** Predictable ecology and geography of West Nile virus transmission in the central United States. *J Vector Ecol*. **2008**:33(2):342–352. doi: 10.3376/1081-1710-33.2.342.

**Poh KC, Chaves LF, Reyna-Nava M, Roberts CM, Fredregill C, Bueno R, Debboun M, Hamer GL.** The influence of weather and weather variability on mosquito abundance and infection with West Nile virus in Harris County, Texas, USA. *Sci Total Environ*. **2019**:675:260–272. doi: 10.1016/j.scitotenv.2019.04.109.

**Porter WT, Barrand ZA, Wachara J, DaVall K, Mihaljevic JR, Pearson T, Salkeld DJ, Nieto NC.** Predicting the current and future distribution of the western black-legged tick, *Ixodes pacificus*, across the Western US using citizen science collections. *PLoS One*. **2021**:16(1):e0244754. doi: 10.1371/journal.pone.0244754.

**Puggioni G, Couret J, Serman E, Akanda AS, Ginsberg HS.** Spatiotemporal modeling of dengue fever risk in Puerto Rico. *Spat Spatiotemporal Epidemiol*. **2020**:35:100375. doi: 10.1016/j.sste.2020.100375.

**Raghavan RK, Goodin DG, Hanzlicek GA, Zolnerowich G, Dryden MW, Anderson GA, Ganta RR.** Maximum entropy-based ecological niche model and bio-climatic determinants of lone star tick (*Amblyomma americanum*) niche. *Vector Borne Zoonotic Dis*. **2016**:16(3): 205–211. doi: 10.1089/vbz.2015.1837.

**Raghavan RK, Goodin DG, Neises D, Anderson GA, Ganta RR.** Hierarchical Bayesian spatio-temporal analysis of climatic and socio-economic determinants of Rocky Mountain spotted fever. *PLoS One*. **2016**:11(3):e0150180. doi: 10.1371/journal.pone.0150180.

**Raghavan RK, Neises D, Goodin DG, Andresen DA, Ganta RR.** Bayesian spatio-temporal analysis and geospatial risk factors of human monocytic ehrlichiosis. *PLoS One*. **2014**:9(7):e100850. doi: 10.1371/journal.pone.0100850.

**Raghavan RK, Peterson AT, Cobos ME, Ganta R, Foley D.** Current and future distribution of the lone star tick, *Amblyomma americanum* (L.) (Acari: Ixodidae) in North America. *PLoS One*. **2019**:14(1):e0209082. doi: 10.1371/journal.pone.0209082.

**Rakotoarinia MR, Blanchet FG, Gravel D, Lapen DR, Leighton PA, Ogden NH, Ludwig A.** Effects of land use and weather on the presence and abundance of mosquito-borne disease vectors in a urban and agricultural landscape in Eastern Ontario, Canada. *PLoS One*. **2022**:17(3):e0262376. doi: 10.1371/journal.pone.0262376.

**Reisen WK, Cayan D, Tyree M, Barker CA, Eldridge B, Dettinger M**. Impact of climate variation on mosquito abundance in California. *J Vector Ecol*. **2008**:33(1):89–98. doi: 10.3376/1081-1710(2008)33[89:iocvom]2.0.co;2.

**Ripoche M, Campagna C, Ludwig A, Ogden NH, Leighton PA.** Short-term forecasting of daily abundance of West Nile virus vectors *Culex pipiens-restuans* (Diptera: Culicidae) and *Aedes vexans* based on weather conditions in southern Québec (Canada). *J Med Entomol*. **2019**:56(3):859–872. doi: 10.1093/jme/tjz002.

**Robert MA, Christofferson RC, Weber PD, Wearing HJ.** Temperature impacts on dengue emergence in the United States: investigating the role of seasonality and climate change. Epidemics. **2019**:28:100344. doi: 10.1016/j.epidem.2019.05.003.

**Robinson SJ, Neitzel DF, Moen RA, Craft ME, Hamilton KE, Johnson LB, Mulla DJ, Munderloh UG, Redig PT, Smith KE, Turner CL, Umber JK, Pelican KM.** Disease risk in a dynamic environment: the spread of tick-borne pathogens in Minnesota, USA. *Ecohealth.* **2015**:12(1):152–163. doi: 10.1007/s10393-014-0979-y.

**Rochlin I, Ninivaggi DV, Hutchinson ML, Farajollahi A**. Climate change and range expansion of the Asian tiger mosquito (*Aedes albopictus*) in Northeastern USA: implications for public health practitioners. *PLoS One*. **2013**:8(4):e60874. doi: 10.1371/journal.pone.0060874.

**Ruybal JE, Kramer LD, Kilpatrick AM.** Geographic variation in the response of *Culex pipiens* life history traits to temperature. *Parasites Vectors*. **2016**:9:116. doi: 10.1186/s13071-016-1402-z.

**Sage KM, Johnson TL, Teglas MB, Nieto NC, Schwan TG.** Ecological niche modeling and distribution of *Ornithodoros hermsi* associated with tick-borne relapsing fever in western North America. *PLoS Negl Trop Dis*. **2017**:11(10):e0006047. doi: 10.1371/journal.pntd.0006047.

**Sánchez-González G, Condé R, Noguez Moreno R, López Vázquez PC.** Prediction of dengue outbreaks in Mexico based on entomological, meteorological and demographic data. *PLoS One*. **2018**:13(8):e0196047. doi: 10.1371/journal.pone.0196047.

**Shocket MS, Verwillow AB, Numazu MG, Slamani H, Cohen JM, El Moustaid F, Rohr J, Johnson LR, Mordecai EA.** Transmission of West Nile and five other temperate mosquito-borne viruses peaks at temperatures between 23°C and 26°C. *Elife*. **2020**:9:e58511. doi: 10.7554/elife.58511.

**Simpson DT, Teague MS, Weeks JK, Lewis AD, D’Addio PM, Moore JD, Thompson JA, Harris AC, Cannella RT, Kaup BZ, Kerscher O, Leu M.** Broad, multi-year sampling effort highlights complex dynamics of the tick-borne pathogen *Ehrlichia chaffeensis* (Rickettsiales: Anaplasmatacae). *J Med Entomol*. **2019**:56(1):162–168. doi: 10.1093/jme/tjy171.

**Skaff NK, Armstrong PM, Andreadis TG, Cheruvelil KS.** Wetland characteristics linked to broad-scale patterns in *Culiseta melanura* abundance and eastern equine encephalitis virus infection. *Parasit* *Vectors*. **2017**:10(1):501. doi: 10.1186/s13071-017-2482-0.

**Skaff NK, Cheng Q, Clemesha RES, Collender PA, Gershunov A, Head JR, Hoover CM, Lettenmaier DP, Rohr JR, Snyder RE, Remais JV.** Thermal thresholds heighten sensitivity of West Nile virus transmission to changing temperatures in coastal California. *Proc Biol Sci*. **2020**:287(1932):20201065. doi: 10.1098/rspb.2020.1065.

**Skaff NK, Cheruvelil KS.** Fine-scale wetland features mediate vector and climate-dependent macroscale patterns in human West Nile virus incidence. *Landsc Ecol*. **2016**:31:1615–1628. doi: 10.1007/s10980-016-0346-1.

**Smith KH, Tyre AJ, Hamik J, Hayes MJ, Zhou Y, Dai L.** Using climate to explain and predict West Nile virus risk in Nebraska. *GeoHealth*. **2020**:4(9):e2020GH000244. doi: 10.1029/2020gh000244.

**Springer YP, Jarnevich CS, Barnett DT, Monaghan AJ, Eisen RJ.** Modeling the present and future geographic distribution of the lone star tick, *Amblyomma americanum* (Ixodida: Ixodidae), in the continental United States. *Am J Trop Med Hyg*. **2015**:93(4):875–890. doi: 10.4269/ajtmh.15-0330.

**St John HK, Adams ML, Masuoka PM, Flyer-Adams JG, Jiang J, Rozmajzl PJ, Stromdahl EY, Richards AL.** Prevalence, distribution, and development of an ecological niche model of *Dermacentor variabilis* ticks positive for *Rickettsia montanensis*. *Vector Borne Zoonotic Dis*. **2016**:16:253–263. doi: 10.1089/vbz.2015.1856.

**Stapp P, Antolin MF, Ball M.** Patterns of extinction in prairie dog metapopulations: plague outbreaks follow El Nino events. *Front Ecol Environ*. **2004**:2(5):235–240. doi: 10.2307/3868263.

**Stephenson C, Coker E, Wisely S, Liang S, Dinglasan RR, Lednicky JA.** Imported dengue case numbers and local climatic patterns are associated with dengue virus transmission in Florida, USA. *Insects*. **2022**:13(2):163. doi: 10.3390/insects13020163.

**Stevens LK, Kolivras KN, Hong Y, Thomas VA, Campbell JB, Prisley SP.** Future Lyme disease risk in the south-eastern United States based on projected land cover. *Geospat Health*. **2019**:14(1). doi: 10.4081/gh.2019.751.

**Tachiiri K, Klinkenberg B, Mak S, Kazmi J.** Predicting outbreaks: a spatial risk assessment of West Nile virus in British Columbia. *Int J Health Geogr*. **2006**:5:21. doi: 10.1186/1476-072x-5-21.

**Tang X, Sedda L, Brown HE.** Predicting eastern equine encephalitis spread in North America: an ecological study. *Curr Res Parasitol Vector Borne Dis*. **2021**:1:100064. doi: 10.1016/j.crpvbd.2021.100064.

**Tran PM, Waller L.** Effects of landscape fragmentation and climate on Lyme disease incidence in the northeastern United States. *Ecohealth*. **2013**:10(4):394–404. doi: 10.1007/s10393-013-0890-y.

**Tran T, Prusinski MA, White JL, Falco RC, Vinci V, Gall WK, Tober K, Oliver J, Sporn LA, Meehan L, Banker E, Backenson PB, Jensen ST, Brisson D.** Spatio-temporal variation in environmental features predicts the distribution and abundance of *Ixodes scapularis*. *Int J Parasit*. **2021**:51(4):311–320. doi: 10.1016/j.ijpara.2020.10.002.

**Uelmen JA, Brokopp C, Patz J.** A 15 year evaluation of West Nile virus in Wisconsin: effects on wildlife and human health. *Int J Environ Res Public Health*. **2020**:17(5):1767. doi: 10.3390/ijerph17051767.

**Uelmen JA, Irwin P, Bartlett D, Brown W, Karki S, Ruiz MO, Fraterrigo J, Li B, Smith RL.** Effects of scale on modeling West Nile virus disease risk. *Am J Trop Med Hyg*. **2021**:104(1):151–165. doi: 10.4269/ajtmh.20-0416.

**Uelmen JA, Irwin P, Brown WM, Karki S, Ruiz MO, Li B, Smith RL.** Dynamics of data availability in disease modeling: an example evaluating the trade-offs of ultra-fine-scale factors applied to human West Nile virus disease models in the Chicago area, USA. *PLoS One*. **2021**:16(5):e0251517. doi: 10.1371/journal.pone.0251517.

**Ukawuba I, Shaman J.** Association of spring-summer hydrology and meteorology with human West Nile virus infection in West Texas, USA, 2002-2016. *Parasit Vectors*. **2018**:11(1):224. doi: 10.1186/s13071-018-2781-0.

**Walsh MG.** The relevance of forest fragmentation on the incidence of human babesiosis: investigating the landscape epidemiology of an emerging tick-borne disease. *Vector Borne Zoonotic Dis*. **2013**:13(4):250–255. doi: 10.1089/vbz.2012.1198.

**Wang G.** Effects of weather and landscape on the equine West Nile virus infection risk in Mississippi, USA. *Geospat Health*. **2015**:10(2):357. doi: 10.4081/gh.2015.357.

**Wang G, Minnis RB, Belant JL, Wax CL.** Dry weather induces outbreaks of human West Nile virus infections. *BMC Infect Dis*. **2010**:10:38. doi: 10.1186/1471-2334-10-38.

**Wang HH, Grant WE, Teel PD.** Simulation of climate-host-parasite-landscape interactions: a spatially explicit model for ticks (Acari: Ixodidae). *Ecol Model*. **2012**:243:42–62. doi: 10.1016/j.ecolmodel.2012.06.007.

**Watson SC, Liu Y, Lund RB, Gettings JR, Nordone SK, McMahan CS, Yabsley MJ.** A Bayesian spatio-temporal model for forecasting the prevalence of antibodies to *Borrelia burgdorferi*, causative agent of Lyme disease, in domestic dogs within the contiguous United States. *PLoS One*. **2017**:12(5):e0174428. doi: 10.1371/journal.pone.0174428.

**Watts MJ, Kotsila P, Mortyn PG, Sarto V, Monteys I, Urzi Brancati C.** Influence of socio-economic, demographic and climate factors on the regional distribution of dengue in the United States and Mexico. *Int J Health Geogr*. **2020**:19(1):44. doi: 10.1186/s12942-020-00241-1.

**Wimberly MC, Baer AD, Yabsley MJ.** Enhanced spatial models for predicting the geographic distributions of tick-borne pathogens. *Int J Health Geogr*. **2008**:7:15. doi: 10.1186/1476-072x-7-15.

**Wimberly MC, Davis JK, Evans MV, Hess A, Newberry PM, Solano-Asamoah N, Murdock CC.** Land cover affects microclimate and temperature suitability for arbovirus transmission in an urban landscape. *PLoS Negl Trop Dis*. **2020**:14(9):e0008614. doi: 10.1371/journal.pntd.0008614.

**Wimberly MC, Hildreth MB, Boyte SP, Lindquist E, Kightlinger L.** Ecological niche of the 2003 West Nile virus epidemic in the northern Great Plains of the United States. *PLoS One*. **2008**:3(12):e3744. doi: 10.1371/journal.pone.0003744.

**Winokur OC, Main BJ, Nicholson J, Barker CM.** Impact of temperature on the extrinsic incubation period of Zika virus in *Aedes aegypti.* *PLoS Negl Trop Dis*. **2020**:14(3):e0008047. doi: 10.1371/journal.pntd.0008047.

**Winters AM, Eisen RJ, Lozano-Fuentes S, Moore CG, Pape WJ, Eisen L.** Predictive spatial models for risk of West Nile virus exposure in eastern and western Colorado. *Am J Trop Med Hyg*. **2008**:79(4):581–590. doi: 10.4269/ajtmh.2008.79.581.

**Witmer FDW, Nawrocki TW, Hahn M.** Modeling geographic uncertainty in current and future habitat for potential populations of *Ixodes pacificus* (Acari: Ixodidae) in Alaska. *J Med Entomol*. **2022**:59(3):976–986. doi: 10.1093/jme/tjac001.

**Yang B, Borgert BA, Alto BW, Boohene CK, Brew J, Deutsch K, DeValerio JT, Dinglasan RR, Dixon D, Faella JM, Fisher-Grainger SL, Glass GE, Hayes R, Hoel DF, Horton A, Janusauskaite A, Kellner B, Kraemer MUG, Lucas KJ, Medina J, Morreale R, Petrie W, Reiner RC, Riles MT, Salje H, Smith DL, Smith JP, Solis A, Stuck J, Vasquez C, Williams KF, Xue RD, Cummings DAT.** Modelling distributions of *Aedes aegypti* and *Aedes albopictus* using climate, host density and interspecies competition. *Plos Neglect Trop Dis*. **2021**:15(3):e0009063. doi: 10.1371/journal.pntd.0009063.

**Yee DA, Ezeakacha NF, Abbott KC.** The interactive effects of photoperiod and future climate change may have negative consequences for a wide-spread invasive insect. *Oikos*. **2017**:126(1):40–51. doi: 10.1111/oik.03635.
