# Supplementary material for: The use of environmental data in descriptive and predictive models of vector-borne disease in North America
Source: J Med Entomol. 2024 Mar 3;61(3):595–602. doi: 10.1093/jme/tjae031 (PMC11078578; doi:10.1093/jme/tjae031)
Supplement: tjae031_suppl_Supplementary_Texts_S2 [file tjae031_suppl_supplementary_texts_s2.docx]

**Supplemental Text S2. Environmental data sources compiled from the included publications.** Links to data sources provided.

**I. Meteorological Data**

- Airport and local weather stations
- ANUSPLIN (<https://fennerschool.anu.edu.au/research/products/anusplin>)
- California Integrated Pest Management program (<http://ipm.ucanr.edu/>)
- California Water Science Center Basin Characterization Model (<https://ca.water.usgs.gov/projects/reg_hydro/basin-characterization-model.html>)
- Cary Institute of Ecosystem Studies (<https://www.caryinstitute.org/>)
- Centers for Disease Control and Prevention (CDC)
  - Wide-ranging Online Data for Epidemiological Research (WONDER) database
- Centro de Recursos Idrisi - México (<http://idrisi.uaemex.mx/>)
- Climatic Research Unit at the University of East Anglia (<https://www.uea.ac.uk/groups-and-centres/climatic-research-unit>)
- CliMond (<https://www.climond.org/>)
- Daymet (<https://daymet.ornl.gov/>)
- Environment Canada (<https://www.canada.ca/en/environment-climate-change.html>)
  - Historical Climate Data (<https://climate.weather.gc.ca/>)
  - Meteorological Service of Canada (<https://weather.gc.ca/>)
  - National Climate Data and Information Archive (<https://climate.weather.gc.ca/>)
- E.V. Smith Research Center, Auburn University (<https://aaes.auburn.edu/regional-centers/>)
- Florida Automated Weather Network (FAWN) (<https://fawn.ifas.ufl.edu/>)
- Illinois State Water Survey (ISWS) (<https://www.isws.illinois.edu/>)
- Maryland Science Center (<https://www.mdsci.org/>)
- Mexican Secretariat of the Environment and Natural Resources
  - Servicio Meteorológico Nacional (Mexican National Meteorological Service) (<https://smn.conagua.gob.mx/es/>)
- Midwestern Regional Climate Center (<https://mrcc.purdue.edu/>)
  - cli-MATE (<https://mrcc.purdue.edu/CLIMATE/>)
- Minnesota State Climatology Office (<https://climateapps.dnr.state.mn.us/index.htm>)
- National Aeronautics and Space Administration (NASA)
  - Ames Research Center Ecological Forecasting Laboratory (<https://www.nasa.gov/ames>)
  - Prediction of Worldwide Energy Resources (POWER) (<https://power.larc.nasa.gov/>)
  - Terrestrial Observation and Prediction System (TOPS) <https://software.nasa.gov/software/ARC-16197-1A>)
- National Center for Atmospheric Research (<https://ncar.ucar.edu/>)
  - Canadian Forest Service BioSIM10 (<https://cfs.nrcan.gc.ca/publications?id=34818>)
- National Oceanic and Atmospheric Administration (NOAA)
  - Climate Data Online (<https://www.ncdc.noaa.gov/cdo-web/datasets>)
  - Climate Prediction Center (<http://cpc.ncep.noaa.gov/>)
  - Dengue Forecasting (<https://dengueforecasting.noaa.gov/>)
  - Global Historical Climatology Network daily/monthly (<https://www.ncei.noaa.gov/products/land-based-station>)
  - Historical Climatology Network (<https://www.ncei.noaa.gov/products/land-based-station>)
  - National Centers for Environmental Information (<https://www.ncei.noaa.gov/>)
  - National Centers for Environmental Prediction (<https://www.weather.gov/ncep/>)
  - National Climatic Data Center (<https://www.ncei.noaa.gov/cdo-web/>)
  - National Weather Service (<https://www.weather.gov/>)
  - North American Regional Reanalysis (<https://www.ncei.noaa.gov/products/weather-climate-models/north-american-regional>)
  - Physical Sciences Laboratory (<https://psl.noaa.gov/>)
    - GHCN CAMS (<https://psl.noaa.gov/data/gridded/data.ghcncams.html>)
- National Water Commissions (CONAGUA) of Mexico (<https://www.gob.mx/conagua>)
- Natural Resources Canada (<https://www.nrcan.gc.ca/>)
- Northwest Climate Science Center Multivariate Adaptive Constructed Analogs (MACA) datasets (<https://climate.northwestknowledge.net/MACA/index.php>)
- PRISM Climate Data (<https://prism.oregonstate.edu/>)
- Royal Netherlands Meteorological Institute’s Climate Explorer (<https://climexp.knmi.nl/start.cgi>)
- Scenarios Network for Alaska and Arctic Planning (<https://uaf-snap.org/>)
- Sistema Nacional de Información del Agua (SINA) (<http://sina.conagua.gob.mx/sina/>)
- TopoWx (<https://www.scrim.psu.edu/resources/topowx/>)
- United States Department of Agriculture (USDA)
  - National Water and Climate Center of the Natural Resources Conservation Service (<https://www.nrcs.usda.gov/wps/portal/wcc/home/>)
  - Natural Resources Conservation Service Snow Telemetry (SNOTEL) Network (<https://data.nal.usda.gov/dataset/snowpack-telemetry-network-snotel>)
  - Wind Erosion and Water Conservation Research: Lubbock, TX ([https://www.ars.usda.gov/plains-area/lubbock-tx/cropping-systems- research-laboratory/wind-erosion-and-water-conservation-research/](https://www.ars.usda.gov/plains-area/lubbock-tx/cropping-systems-research-laboratory/wind-erosion-and-water-conservation-research/))
- Weather Underground (<https://www.wunderground.com/>)
- Western Regional Climate Center (<https://wrcc.dri.edu/>)
- World Bank Group Climate Change Knowledge Portal (<https://climateknowledgeportal.worldbank.org/>)
- WorldClim (<https://worldclim.org/>)
- World Weather Online (<https://www.worldweatheronline.com/>)

**II. Elevation Data**

- ASTER Global Digital Elevation Map (<https://asterweb.jpl.nasa.gov/gdem.asp>)
- County Highpointers Association (<http://www.cohp.org/>)
- Daymet (<https://daymet.ornl.gov/>)
- Digital Elevation Model (DEM) for the state of Florida (<https://www.fgdl.org/metadata/fgdl_html/flidar_mosaic_m.htm>)
- Earth Resources Data Analysis System (<https://gisgeography.com/erdas-imagine/>)
- Earth Resources Observations and Science (EROS) Center (<https://www.usgs.gov/centers/eros>)
  - Hydro 1K (<https://www.usgs.gov/centers/eros/science/usgs-eros-archive-digital-elevation-hydro1k>)
- National Aeronautics and Space Administration (NASA)
  - Shuttle Radar Topography Mission (SRTM) (<https://www2.jpl.nasa.gov/srtm/>)
- Natural Resources Canada Digital Elevation Model (<https://open.canada.ca/data/en/dataset/7f245e4d-76c2-4caa-951a-45d1d2051333>)
- PRISM Climate Data (<https://prism.oregonstate.edu/>)
- United States Geological Survey (USGS) (<https://www.usgs.gov/>)
  - Digital Elevation Models (<https://www.usgs.gov/3d-elevation-program/about-3dep-products-services>)
  - GTOPO30 Digital Elevation Model (<https://www.usgs.gov/centers/eros/science/usgs-eros-archive-digital-elevation-global-30-arc-second-elevation-gtopo30>)
  - National Elevation Dataset (<https://www.sciencebase.gov/catalog/folder/4fcf8fd4e4b0c7fe80e81504>)
  - National Map (<https://www.usgs.gov/programs/national-geospatial-program/national-map>)

**III. Future Weather/Simulations**

- California Water Science Center Basin Characterization Model (<https://ca.water.usgs.gov/projects/reg_hydro/basin-characterization-model.html>)
- Canadian Centre for Climate Modelling and Analysis climate models (<https://www.canada.ca/en/environment-climate-change/services/climate-change/science-research-data/modeling-projections-analysis/centre-modelling-analysis/models.html>)
- Canadian Seasonal to Interannual Prediction System Version 2 (CanSIPv2) (<https://journals.ametsoc.org/view/journals/wefo/35/4/wafD190259.xml>)
- Centre National de Recherches Météorologiques (<http://www.umr-cnrm.fr/?lang=fr>)
  - CNRM-CM5 (<https://www.umr-cnrm.fr/spip.php?article126&lang=en>)
- Centre pour l’Étude et la Simulation du Climat à l’Échelle Régionale (ESCER) (<https://escer.uqam.ca/>)
- Climate Change, Agriculture, and Food Security Climate Data Portal (<http://ccafs-climate.org/>)
- Climate Model Diagnosis and Intercomparison (<https://pcmdi.llnl.gov/index.html>)
  - Coupled Model Intercomparison Project Phase 5 (CMIP5) (<https://pcmdi.llnl.gov/mips/cmip5/>)
- Commonwealth Scientific and Industrial Research Organization (CSIRO) (<https://data.csiro.au/browse/kw/GCM>)
- Community Climate System Model version 4 (<https://geomodeling.njnu.edu.cn/modelItem/2b7c21d0-1f27-42aa-a4d7-f6ca2220d02e>)
- Consultative Group on International Agricultural Research (CGIAR) (<https://www.cgiar.org/>)
- Coordinated Regional Climate Downscaling Experiment (CORDEX) project (<https://cordex.org/>)
- Danish Meteorological Institute (<https://www.dmi.dk/>)
  - HIRHAM5
- European Centre for Medium-Range Weather Forecasts Reanalysis (<https://www.ecmwf.int/>)
- High-Resolution WRF Simulations of the Current and Future Climate of North America (<https://rda.ucar.edu/datasets/ds612.0/>)
- Intergovernmental Panel on Climate Change assessments (<https://www.ipcc-data.org/>)
  - Representative Concentration Pathways
- International Center for Tropical Agriculture (<https://wle.cgiar.org/content/international-center-tropical-agriculture-ciat>)
- Inter-Sectoral Impact Model Intercomparison Project (<https://www.isimip.org/>)
  - MIROC5 (<https://www.isimip.org/gettingstarted/input-data-bias-adjustment/details/21/>)
- Instituto Nacional de Ecología y Cambio Climático (<https://www.gob.mx/inecc>)
- Irish Centre for High-End Computing EC Earth climate model (<https://www.ichec.ie/>)
- Long Ashton Research Station - Weather Generator (LARS-WG) (<https://sites.google.com/view/lars-wg/>)
- Max Planck Institute for Meteorology (MPI-M) European Centre Hamburg global climate model (<https://mpimet.mpg.de/en/science/models/mpi-esm/echam>)
- Met Office
  - HadCM3 (<https://www.metoffice.gov.uk/research/approach/modelling-systems/unified-model/climate-models/hadcm3>)
  - HadGEM2(<https://www.metoffice.gov.uk/research/approach/modelling-systems/unified-model/climate-models/hadgem2>)
- Model for Interdisciplinary Research on Climate version 5 (MIROC5) (<https://glisaclimate.org/node/2539>)
- National Aeronautics and Space Administration (NASA) (<https://www.nasa.gov/>)
  - Atmosphere-Ocean General Circulation Model (AOGCM) (<https://gmao.gsfc.nasa.gov/products/climateforecasts/GEOS5/DESC/aogcm.php>)
  - Goddard Institute for Space Studies (<https://data.giss.nasa.gov/>)
- National Center for Atmospheric Research
  - Community Climate System Model version 3 (CCSM3) (<https://www.cgd.ucar.edu/projects/trace>)
  - Parallel Climate Model (<https://www2.cgd.ucar.edu/pcm/>)
- National Oceanic and Atmospheric Administration (<https://www.noaa.gov/>)
  - Geophysical Fluid Dynamics Laboratory models (<https://data1.gfdl.noaa.gov/>)
- North American Regional Climate Change Assessment Program (<https://www.narccap.ucar.edu/>)
- Second Generation Canadian Earth System Model (CanESM2) (<https://www.canada.ca/en/environment-climate-change/services/climate-change/science-research-data/modeling-projections-analysis/centre-modelling-analysis/models/second-generation-earth-system-model.html>)
- Shared Socioeconomic Pathways (SSPs) (<https://secure.iiasa.ac.at/web-apps/ene/SspDb/dsd?Action=htmlpage&page=about>)
- Swedish Meteorological and Hydrological Institute (<https://www.smhi.se/en>)
  - RCA4
- United States Geological Survey (USGS) Land Cover Projections (<https://www.usgs.gov/centers/eros/science/land-cover-projections>)
- Weather Research and Forecasting (<https://www.mmm.ucar.edu/models/wrf>)

**IV. Satellite Data**

- Global 1 km Consensus Land Cover data set (<https://www.earthenv.org/landcover>)
- Global Land Cover Facility (GLCF) (<https://geog.umd.edu/feature/global-land-cover-facility-%28glcf%29>)
- Google Earth (<https://earth.google.com/web/>)
- Manitoba Remote Sensing Centre (MRSC) (<https://www.gov.mb.ca/geomb/geospatial-data-acquisition/index.html>)
- MERRAclim (<https://www.nature.com/articles/sdata201778>)
- Multivariate Adaptive Constructed Analogs (MACA) (<https://www.climatologylab.org/maca.html>)
- National Aeronautics and Space Administration (NASA)
  - Goddard Earth Sciences Data and Information Services Center (<https://www.earthdata.nasa.gov/eosdis/daacs/gesdisc>)
    - Advanced Microwave Scanning Radiometer (AMSR)
    - National Land Data Assimilation System (NLDAS)
    - Visible Infrared Scanner (VIRS)
  - Goddard Space Flight Center
    - Normalized Difference Vegetation Index (<https://modis-land.gsfc.nasa.gov/vi.html>)
    - Tropical Rainfall Measuring Mission (<https://trmm.gsfc.nasa.gov/>)
  - Landsat Thematic Mapper (<https://landsat.gsfc.nasa.gov/thematic-mapper/>)
    - Multispectral Scanner System (<https://landsat.gsfc.nasa.gov/multispectral-scanner-system/>)
  - Moderate Resolution Imaging Spectroradiometer (MODIS) (<https://modis.gsfc.nasa.gov/>)
  - Prediction of Worldwide Energy Resources (POWER (<https://power.larc.nasa.gov/>)
- National Oceanic and Atmospheric Administration (NOAA)
  - Advanced Very High Resolution Radiometer (AVHRR) (<https://earthexplorer.usgs.gov/>)
  - Defense Meteorological Satellite Program (<https://data.noaa.gov/dataset/dataset/defense-meteorological-satellite-program-dmsp>)
  - North American Multi-Model Ensemble (NMME) (<https://www.ncei.noaa.gov/products/weather-climate-models/north-american-multi-model>)
- United States Geological Survey (USGS)
  - Early Warning and Environmental Monitoring Program (EWEM) (<https://earlywarning.usgs.gov/>)
  - Landsat 7 Enhanced Thematic Mapper (ETM+) (<https://www.usgs.gov/landsat-missions/landsat-7>)
  - Landsat 5 ™ imagery from the Global Visualiation Viewer (<https://glovis.usgs.gov/>)
  - Land Processes Distributed Active Archive Center (LP DAAC) (<https://lpdaac.usgs.gov/>)

**V. Topographic Data**

- Agriculture and Agri-Use Canada (AAFC) Annual Crop Inventory (<https://open.canada.ca/data/en/dataset/ba2645d5-4458-414d-b196-6303ac06c1c9>)
- California Department of Forestry and Fire Protection Land Cover Mapping and Monitoring Program (<https://frap.fire.ca.gov/mapping/>)
- California Integrated Pest Management (<https://ipm.ucanr.edu/>)
- Canada’s National Forest Inventory (NFI) (<https://nfi.nfis.org/en/>)
- Comisión Nacional para el Conocimiento y Uso de la Biodiversidad (CONABIO) (<https://www.gob.mx/conabio>)
- Ducks Unlimited Canada (DUC) Canadian Wetland Inventory (<https://www.ducks.ca/initiatives/canadian-wetland-inventory/>)
- Florida Geographic Data Library (FGDL) (<https://fgdl.org/>)
- Government of British Columbia Terrain Resource Information Management (TRIM) (<https://www2.gov.bc.ca/gov/content/data/geographic-data-services/topographic-data/terrain>)
- Information Services Corporation of Saskatchewan (<https://www.isc.ca/Pages/default.aspx>)
- Instituto Nacional de Estadística y Geografía (INEGI) (<https://www.inegi.org.mx/temas/edafologia/>)
- International Soil Reference and Information Centre’s SoilGrids Global Dataset (<https://dev.isric.org/explore/soilgrids>)
- Manitoba Land Inititative Data Warehouse (<https://mli2.gov.mb.ca/mli_data/index.html>)
- Ministère du Développement durable, de l’Environnement, de la Faune et des Parcs Québec (<https://www.environnement.gouv.qc.ca/>)
- Minnesota Department of Natural Resources (<https://www.dnr.state.mn.us/>)
- National Hydrology Database Plus v2 (<https://nhdplus.com/NHDPlus/>)
- Natural Resources Canada (<https://www.nrcan.gc.ca/home>)
  - Government of Canada’s Topographic Information (<https://www.nrcan.gc.ca/science-and-data/science-and-research/earth-sciences/geography/topographic-information/10785>)
  - Land cover map (<https://www.nrcan.gc.ca/maps-tools-and-publications/21448>)
  - National Hydrographic Network (<https://www.nrcan.gc.ca/science-and-data/science-and-research/earth-sciences/geography/topographic-information/geobase-surface-water-program-geeau/national-hydrographic-network/21361>)
- New Brunswick Hydrographic Network (<http://www.snb.ca/geonb1/e/index-E.asp>)
- NYS GIS Clearinghouse (<https://gis.ny.gov/gisdata/>)
- Ontario Ministry of Natural Resources and Forestry (<https://www.ontario.ca/page/ministry-natural-resources-and-forestry>)
- Soils of Canada (<https://soilsofcanada.ca/>)
- Topologically Integrated Geographic Encoding and Referencing (TIGER) (<https://tigerweb.geo.census.gov/tigerwebmain/TIGERweb_main.html>)
- United States Census Bureau (<https://www.census.gov/>)
- United States Department of Agriculture (<https://www.usda.gov/>)
  - Agricultural Research Service (<https://www.ars.usda.gov/>)
  - Cropland Data Layer (<https://data.nal.usda.gov/dataset/cropscape-cropland-data-layer>)
  - Forest Service’s Forest Inventory and Analysis (<https://www.fia.fs.usda.gov/>)
  - FRAGSTATS (<https://www.fs.usda.gov/research/treesearch/3064>)
  - National Resources Conservation Service
    - Soil data mart (<https://websoilsurvey.sc.egov.usda.gov/App/HomePage.htm>)
    - Soil Survey Geographic Database (<https://www.nrcs.usda.gov/resources/data-and-reports/soil-survey-geographic-database-ssurgo>)
  - United States General Soil Map (<https://data.nal.usda.gov/dataset/united-states-general-soil-map-statsgo2>)
- United States Environmental Protection Agency
  - EnviroAtlas Meter-Scale Urban Land Cover (MULC) (<https://cfpub.epa.gov/si/si_public_record_report.cfm?Lab=CPHEA&dirEntryId=349211>)
- United States Fish and Wildlife Service National Wetlands Inventory (<https://www.fws.gov/program/national-wetlands-inventory>)
- United States Geological Survey (USGS)
  - Earth Resources Observations and Science (EROS) Center (<https://www.usgs.gov/centers/eros>)
    - Global Visualization Viewer (<https://glovis.usgs.gov/>)
    - Hydro 1K (<https://www.usgs.gov/centers/eros/science/usgs-eros-archive-digital-elevation-hydro1k>)
  - Gap Analysis Program (<https://www.usgs.gov/programs/gap-analysis-project>)
  - Geochemical and Mineralogical Data for Soils of the Conterminous United States (<https://pubs.usgs.gov/ds/801/>)
  - Global Land Cover Characteristics Database (<https://www.usgs.gov/centers/eros/science/usgs-eros-archive-land-cover-products-global-land-cover-characterization-glcc#overview>)
  - Land Cover Trends Project (<https://www.usgs.gov/centers/western-geographic-science-center/science/land-cover-trends>)
  - Land Use Land Cover FORE-SCE Model (<https://www.usgs.gov/special-topics/land-use-land-cover-modeling/land-cover-modeling-methodology-fore-sce-model>)
  - National Hydrography Dataset (<https://www.usgs.gov/national-hydrography/national-hydrography-dataset>)
  - National Land Cover Database (<https://www.usgs.gov/centers/eros/science/national-land-cover-database>)
  - Physiologic Provinces (<https://water.usgs.gov/GIS/metadata/usgswrd/XML/mrb_e2rf1_physio.xml>)
- Université de Montréal (UdeM) (<https://www.umontreal.ca/en/>)
- Wisconsin Department of Natural Resources Wiscland 2 (<https://www.sco.wisc.edu/2016/09/23/wiscland-2-project-complete-data-now-available/>)

**VI. Additional Data**

Daylight

- Astonomical Applications Department of the United States Naval Observatory (<https://aa.usno.navy.mil/>)
- Daymet (<https://daymet.ornl.gov/>)

Drought

- US Drought Monitor (<https://droughtmonitor.unl.edu/>)
- National Climatic Data Center (<https://www.ncei.noaa.gov/cdo-web/>)
- National Oceanic and Atmospheric Administration Climate Data Online (CDO) (<https://www.ncei.noaa.gov/cdo-web/>)
- Variable Infiltration Capacity (VIC) Model (<https://vic.readthedocs.io/en/master/>)
- WestWide Drought Tracker (<https://www.drought.gov/data-maps-tools/westwide-drought-tracker-wwdt-gridded-monthly-drought-indices-western-us>)

El Niño-Southern Oscillation (ENSO)

- Southern Oscillation Index (<https://www.ncei.noaa.gov/access/monitoring/enso/soi>)

Hurricane

- National Oceanic and Atmospheric Administration (NOAA) (<https://www.noaa.gov/>)

Oceanographic

- Institute for Marine Remote Sensing (<https://imars.usf.edu/>)
